# Genetic interactions between specific chromosome copy number alterations dictate complex aneuploidy patterns

**DOI:** 10.1101/gad.319400.118

**Published:** 2018-12-01

**Authors:** Madhwesh C. Ravichandran, Sarah Fink, Matthew N. Clarke, Franziska Christina Hofer, Christopher S. Campbell

**Affiliations:** Department of Chromosome Biology, Max F. Perutz Laboratories, University of Vienna, Vienna Biocenter, Vienna 1030, Austria

**Keywords:** chromosomes, aneuploidy, chromosomal instability, CIN, adaptation, chromosomal passenger complex

## Abstract

Cells that contain an abnormal number of chromosomes are called aneuploid. High rates of aneuploidy in cancer are correlated with an increased frequency of chromosome missegregation, termed chromosomal instability (CIN). Both high levels of aneuploidy and CIN are associated with cancers that are resistant to treatment. Although aneuploidy and CIN are typically detrimental to cell growth, they can aid in adaptation to selective pressures. Here, we induced extremely high rates of chromosome missegregation in yeast to determine how cells adapt to CIN over time. We found that adaptation to CIN occurs initially through many different individual chromosomal aneuploidies. Interestingly, the adapted yeast strains acquire complex karyotypes with specific subsets of the beneficial aneuploid chromosomes. These complex aneuploidy patterns are governed by synthetic genetic interactions between individual chromosomal abnormalities, which we refer to as chromosome copy number interactions (CCNIs). Given enough time, distinct karyotypic patterns in separate yeast populations converge on a refined complex aneuploid state. Surprisingly, some chromosomal aneuploidies that provided an advantage early on in adaptation are eventually lost due to negative CCNIs with even more beneficial aneuploid chromosome combinations. Together, our results show how cells adapt by obtaining specific complex aneuploid karyotypes in the presence of CIN.

## Abstract (precis)

In this study, Ravichandran et al. induced extremely high rates of chromosome missegregation in yeast to determine how cells adapt to chromosomal instability (CIN) over time. They show that adaptation to CIN initially occurs through many different individual chromosomal aneuploidies, and their findings provide new insights into how cells adapt by obtaining specific complex aneuploid karyotypes in the presence of CIN.

Accurate distribution of replicated genetic material to daughter cells is one of the most fundamental requirements of cell division. Errors in chromosome segregation lead to the loss or gain of chromosomes, a state called aneuploidy. In many cases, cancer cells have highly aberrant chromosome copy numbers and extremely complex karyotypes (Mitelman Database of Chromosome Aberrations and Gene Fusions in Cancer, http://cgap.nci.nih.gov/Chromosomes/Mitelman). The complexity of these cancer karyotypes makes it difficult to retroactively piece together the steps in their formation.

Aneuploidy is generally associated with decreased cellular fitness. Experiments using yeast or human cell lines engineered with specific aneuploidies have revealed that doubling the copy number of single chromosomes leads to increased expression of nearly all of the genes on that chromosome (Torres et al. 2007; Stingele et al. 2012). This creates imbalances in the expression levels between genes on different chromosomes. These imbalances lead to proteotoxic stress and increased rates of mutation and chromosome missegregation (Sheltzer et al. 2011; Oromendia et al. 2012; Zhu et al. 2012; Passerini et al. 2016).

In contrast, in certain cases, aneuploidy provides a selective advantage. Specific aneuploidies have been shown to provide resistance to stress conditions in yeast (Selmecki et al. 2006; Chen et al. 2012). Aneuploidy can also act as a suppressor of certain mutations (Rancati et al. 2008; Liu et al. 2015; Ryu et al. 2016). Although aneuploid chromosomes alter the stoichiometry of many genes, the selective advantage can often be attributed to the change in expression of one or two genes (Hughes et al. 2000; Rancati et al. 2008; Ryu et al. 2016). This indicates that, similar to other types of mutations, aneuploidy is typically detrimental but can be beneficial to cellular growth in specific cases. These results also suggest that the selective force on aneuploidy for a particular chromosome is determined mainly by the specific advantage of altered expression of a few genes. These advantages are tempered by the general disadvantage of expression imbalances for many other genes.

Aneuploidy arises from errors in cell division that result in the uneven distribution of the chromosomes between the two daughter cells. The increased rate of the formation of aneuploidy resulting from chromosome missegregation errors is called chromosomal instability (CIN). Importantly, both aneuploidy and CIN are hallmarks of cancer and are indicators of poor prognosis (for review, see Sansregret et al. 2018). Some cancer cell lines have chromosome segregation errors as high as 1% per chromosome per cell division, which would be detrimental to the growth of normal cells (Thompson and Compton 2008). Little is known about how cancer cells adapt to thrive with high levels of CIN.

In this study, we induced extremely high rates of chromosome missegregation in yeast to determine how they adapt to CIN over time. We found that the yeast adapted primarily through the accumulation of beneficial aneuploidies of many different chromosomes. The adapted yeast acquired complex karyotypes that consisted of specific subsets of the beneficial aneuploid chromosomes. By engineering the observed complex aneuploidy patterns in the absence of CIN, we demonstrate that distinct patterns of complex karyotypes are created by genetic interactions between individual aneuploid chromosomes. Given enough time to adapt, divergent complex karyotype patterns eventually converge on an “optimal” complex karyotype that maximizes the selective advantage of individual chromosomal aneuploidies while minimizing the negative genetic interactions between aneuploidies. This process often involves the loss of beneficial aneuploidies in order to gain other, incompatible aneuploid chromosomes. Overall, our results show that complex aneuploid karyotypes result from CIN in a stepwise manner that is heavily influenced by genetic interactions between aneuploid chromosomes.

## Results

### Yeast cells adapt to CIN through frequent accumulation of specific aneuploidies

**Figure 1.**
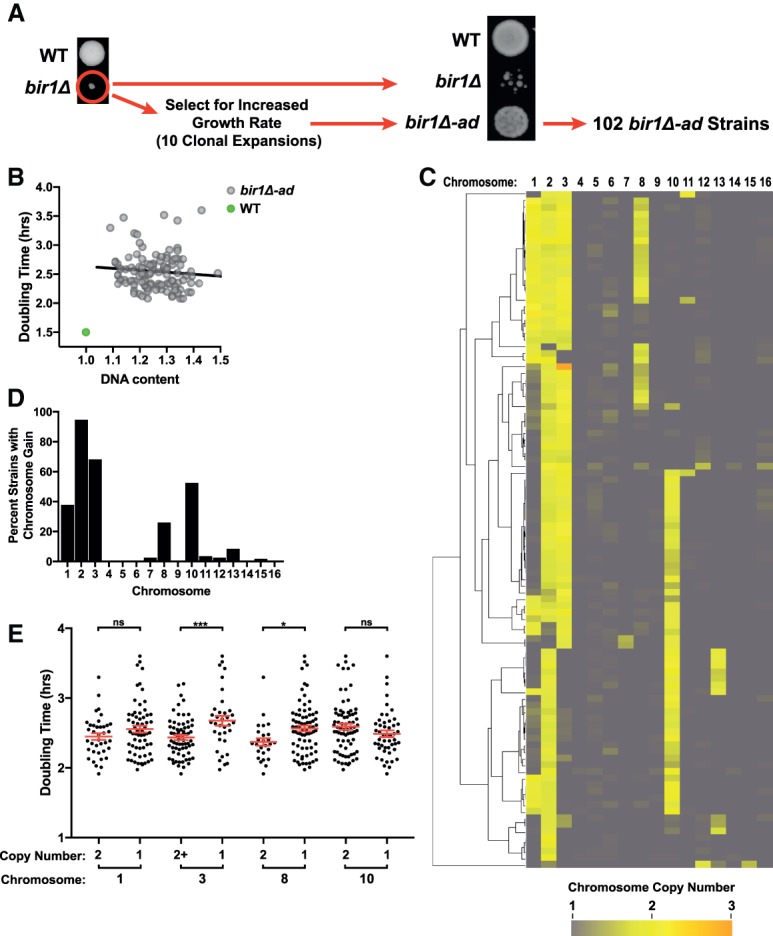
Adaptation to *BIR1* deletion generates complex aneuploid karyotypes. (*A*) Schematic for the generation of 102 strains adapted to *BIR1* deletion (*bir1Δ-ad*). At the *left*, colonies from wild-type and *bir1Δ* spores are shown 4 d after tetrad dissection of a *BIR1*/ *bir1Δ* diploid onto rich (YPA plus 2% dextrose [YPAD]) medium. The *bir1Δ* cells were then grown for ∼200 generations through 10 clonal expansions. At the *right*, equal optical densities of a wild-type strain, a *bir1Δ* strain, and its corresponding *bir1Δ-ad* strain were spotted on YPAD plates. (*B*) Lack of correlation between growth rates and degree of aneuploidy for the *bir1Δ-ad* strains. Doubling times in liquid YPAD of all 102 *bir1Δ-ad* strains were measured by optical density and plotted against the DNA content as measured by flow cytometry. Pearson's correlation coefficient = −0.09. Two-tailed *P*-value = 0.38. (*C*) Heat map visualization of chromosome copy number data for the *bir1Δ-ad* strains as measured by read counts from whole-genome sequencing. Each of the 102 *bir1Δ-ad* strains is represented as a row and clustered to show groupings of karyotype patterns. (*D*) Frequency of aneuploidy for each chromosome in the haploid *bir1Δ-ad* strains based on binarization of the data in *C*. (*E*) Comparison of the growth rates of haploid *bir1Δ-ad* strains with and without extra copies of chromosomes 1, 3, 8, and 10. The mean doubling time and the standard error of each group are shown. (*) *P* ≤ 0.01; (***) *P* ≤ 0.0001; (ns) not significant, unpaired *t*-test.

To induce high rates of CIN in haploid budding yeast, we deleted the Survivin homolog Bir1. Bir1 is a member of the chromosomal passenger complex (CPC), which prevents chromosome missegregation by detecting and correcting improper connections between chromosomes and the mitotic spindle. Following tetrad dissection of a *BIR1*/*bir1Δ* heterozygous diploid, only ∼10% of the *bir1Δ* spores are able to form colonies (Sandall et al. 2006). The cells in the surviving 10% of colonies have extremely high rates of chromosome missegregation, making *bir1Δ* a strong candidate for inducing complex aneuploidy (Campbell and Desai 2013). To obtain strains in a semistable state amenable to further analysis, >100 isolated cells from 19 individual *bir1Δ* spores were allowed to adapt over 10 clonal expansions by selecting a single colony every 2 d. These haploid adapted strains, now called *bir1Δ-ad,* grow faster than the *bir1Δ* strains prior to adaptation; however, their growth rates remain lower than that of wild-type strains (Fig. 1A,B). To directly measure the rate of chromosome missegregation of the *bir1Δ* strains after adaptation, we monitored a fluorescently labeled chromosome by live microscopy. The missegregation rate of chromosome 4 for a subset of the *bir1Δ-ad* strains was found to vary from 0.5% to 4.2% per cell division (Supplemental Fig. S1A). In contrast, chromosome missegregation rates for four unadapted strains were significantly higher, ranging from 2.8% to 7.7% (*P* = 0.0013). As an additional assay for CIN, we measured the growth of the *bir1Δ-ad* strains on plates with a moderate amount of the microtubule-depolymerizing drug benomyl (10 µg/mL). The adapted strains maintained strong sensitivity to the drug (Supplemental Fig. S1B,C). Taken together, the above results demonstrate the adapted *bir1Δ* yeast display a partial decrease in CIN rates yet maintain a strong CIN phenotype.

To determine the degree of aneuploidy in the adapted strains, the DNA content of the *bir1Δ-ad* strains was measured by flow cytometry. All 102 adapted strains had substantially increased DNA content, with increases ranging from 10% to 40% over wild type, demonstrating large amounts of aneuploidy consistent with approximately one to six extra chromosomes in each strain. Although the *bir1Δ-ad* strains display a remarkable heterogeneity in both growth rate and degree of aneuploidy, there was no clear correlation between the two (Fig. 1B). This lack of correlation indicates that there is not a simple relationship between the induced CIN, the resultant aneuploidy, and their impact on cellular fitness.

To determine the degree to which the growth defects in the *bir1Δ-ad* strains result from either ongoing CIN (from the continued lack of Bir1) or the resulting aneuploidy, we added the *BIR1* gene back into the adapted strains via single-copy insertions. *BIR1* add-back fully rescued the benomyl sensitivity for nearly all of the adapted strains, demonstrating a rescue of the CIN phenotype (Supplemental Fig. S1B,C). The growth rates of the add-back strains only partially recovered with readdition of the *BIR1* gene, indicating that aneuploidy also contributes directly to the growth defects in the *bir1Δ-ad* strains (Supplemental Fig. S1D). Most of the *bir1Δ-ad* strains had decreased levels of aneuploidy following *BIR1* add-back (Supplemental Fig. S1E), suggesting that the absence of *BIR1* was an ongoing source of selection for specific aneuploidies. To specifically assess the effect of aneuploidy on the cellular fitness, we analyzed only those strains that maintained similar levels of aneuploidy after *BIR1* add-back (28 out of 102 *bir1Δ-ad* strains) (Supplemental Fig. S1E). These aneuploid *BIR1* add-back strains showed a partial (∼50% median) rescue in growth (Supplemental Fig. S1F). We conclude from these results that the growth defects in the *bir1Δ-ad* strains are caused by a combination of ongoing CIN as well as the resulting aneuploidy.

### Disomy of different chromosomes can contribute directly to CIN adaptation

For detailed determination of the types of aneuploidy acquired in the adapted strains, their genomes were sequenced, and the relative chromosome copy numbers were determined from the read counts. The *bir1Δ-ad* strains frequently had extra copies of chromosomes 1, 2, 3, 8, and 10 (traditionally referred to as the roman numerals I, II, III, VIII, and X in yeast), each of which was seen in over a quarter of the strains (Fig. 1C,D). No chromosome rearrangements or large insertions or deletions were present in any of the adapted strains. Although nearly all of the strains acquired point mutations during adaptation (average of approximately two mutations per strain), the nonsynonymous mutations in coding regions were not significantly enriched for genes reported to be involved in CIN (*P* = 0.53). Of the genes mutated in *bir1Δ-ad* strains, 15.8% (30 of 190 genes) (Supplemental Table S3) were CIN genes, which is similar to the 14.5% of genes in the yeast genome (874 of 6000 genes). Additionally, no gene ontology (GO) terms were significantly enriched for in the list of genes mutated in the *bir1Δ-ad* strains (false discovery rate [FDR] < 0.05). We additionally identified 41 heterozygous gene mutations on disomic chromosomes (Supplemental Table S4). These mutations were also not significantly enriched for GO terms. The above result suggests that improved growth from adaptation was generally not a result of mutations in certain genes. Together, these results point to aneuploidy being the most substantial genomic alteration in the *bir1Δ-ad* strains.

**Figure 2.**
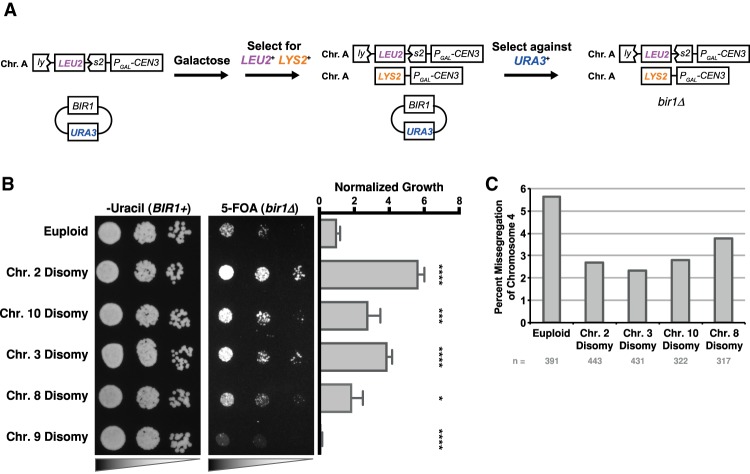
Disomy of specific chromosomes provides an initial advantage to *BIR1* deletion. (*A*) Schematic of a galactose-inducible system to engineer disomy of specific chromosomes prior to *BIR1* deletion. Strains transformed with a construct (*P_GAL_CEN3 lys2::LEU2*) and a minichromosome containing *BIR1* and *URA3* were grown in medium containing galactose to induce chromosome nondisjunction during cell division. Disomy of the desired chromosome was then selected for on minimal medium plates lacking leucine and lysine. Disomic strains for the chromosome of interest were grown on plates containing the drug 5-FOA to select for loss of the *URA3* gene, creating *bir1Δ* yeast that are disomic for the chromosome of interest. (*B*) Tenfold dilution series of disomic strains on minimal medium plates with 5-FOA (selecting against *URA3*) or lacking uracil (selecting for *URA3*) plates. The graph shows the quantification of the growth of each strain after selection against *BIR1* with 5-FOA. All values were normalized to the initially euploid strain. (*) *P* ≤ 0.05; (***) *P* ≤ 0.0001; (****) *P* ≤ 0.00001, unpaired *t*-test. (*C*) Missegregation rates of GFP-labeled chromosome 4 for *bir1Δ* strains from 5-FOA plates as in *B*. The total numbers of quantified segregation events (*n*) are indicated *below* the graph. See Supplemental Figure S1A for examples of segregation and missegregation events.

To determine the degree to which the growth benefits from adaptation could be attributed to certain types of aneuploidy, we compared the doubling times of *bir1Δ-ad* strains with and without gains of chromosomes 1, 3, 8, and 10. There was an insufficient number of strains without chromosome 2 aneuploidy (six out of 102) to make a meaningful comparison (Fig. 1C). Adapted strains with gains of chromosomes 3 or 8 had significantly improved fitness over those that carry only one copy of those chromosomes (Fig. 1E). Chromosome 1 disomy was correlated with a small insignificant increase in fitness (*P* = 0.14). Surprisingly, disomy of chromosome 10 was not correlated with any increase in fitness despite over half of the adapted *bir1Δ* strains containing an extra copy of chromosome 10 (Fig. 1D,E). This discrepancy could be explained by one of the following: (1) Disomy of chromosome 10 in the background of *BIR1* deletion is fitness-neutral, in which case, over a long enough period of time, half of the strains would be expected to have one copy, and half would have two, or (2) disomy of chromosome 10 provides an initial adaptive advantage that decreases with time. To test this second possibility, we engineered strains with individual disomies prior to removal of the *BIR1* gene (Fig. 2A). Aneuploidy was induced via expression of a strong centromere-proximal galactose-inducible promoter and selected for by stochastic recombination events that restore the function of a selectable marker (Anders et al. 2009). *BIR1* was subsequently lost by counterselection of the plasmid-linked *URA3* gene with the drug 5-fluoroorotic acid (5-FOA) (Fig. 2A). Strains with disomy of chromosomes 2, 3, 8, and 10 had significantly increased growth relative to the euploid control on 5-FOA plates selecting for *bir1Δ* mutants (Fig. 2B). In contrast, a strain with disomy of chromosome 9, which was never observed in the adapted strains, had drastically impaired growth in the absence of *BIR1*. All five strains grew similarly on nonselective (*BIR1*^+^) plates. The increase in initial fitness following *bir1Δ* for the beneficial aneuploidies corresponded with a decrease in the missegregation rate of chromosome 4, indicating that disomy of these chromosomes partially suppresses the CIN phenotype (Fig. 2C). We conclude that high frequencies of certain chromosome gains in adapted *bir1Δ* strains, including chromosome 10, result from an initial benefit in fitness shortly after CIN initiation.

Chromosome 2 disomy had the strongest rescue of the *bir1Δ* phenotype and was the most frequent aneuploidy in the *bir1Δ-ad* strains, indicating that there is a gene or set of genes on that chromosome that contributes strongly to survival following *BIR1* deletion. Notably, the gene for the CPC subunit Sli15 is on chromosome 2. We therefore put an additional copy of *SLI15* on chromosome 5 to determine whether it would partially rescue the *bir1Δ* phenotype (Supplemental Fig. S2A). *SLI15* duplication had levels of growth similar to chromosome 2 disomy immediately following *BIR1* deletion (Supplemental Fig. S2B). Furthermore, adapted *bir1Δ* strains with *SLI15* duplication had drastically reduced amounts of chromosome 2 disomy and significantly improved growth rates (Supplemental Fig. S2C,D). Sequencing of 12 *SLI15-*duplicated *bir1Δ-ad* strains showed that these strains still accumulated the other four frequent disomies seen in the original *bir1Δ-ad* strains (chromosomes 1, 3, 8, and 10) when chromosome 2 disomy is absent, demonstrating that the other four disomies are selected for independently of chromosome 2 (Supplemental Fig. S2E). Attempts to create *bir1Δ* strains with the sole copy of *SLI15* on chromosome 5 failed to produce any viable spores. This indicates that *SLI15* duplication is an essential initial step in adaptation to *BIR1* deletion and is not possible on a chromosome whose disomy is associated with severe growth defects (Torres et al. 2007). We therefore relocated *SLI15* as the only copy on a chromosome that we knew could be duplicated: chromosome 8. None of the adapted relocated *SLI15* strains had disomy of chromosome 2 (zero out of seven strains) (Supplemental Fig. S2F,G), further demonstrating that increased expression of Sli15 is the sole basis behind the frequent disomy of chromosome 2 in *bir1Δ-ad* strains.

### Positive and negative correlations occur between chromosome copy number alterations

**Figure 3.**
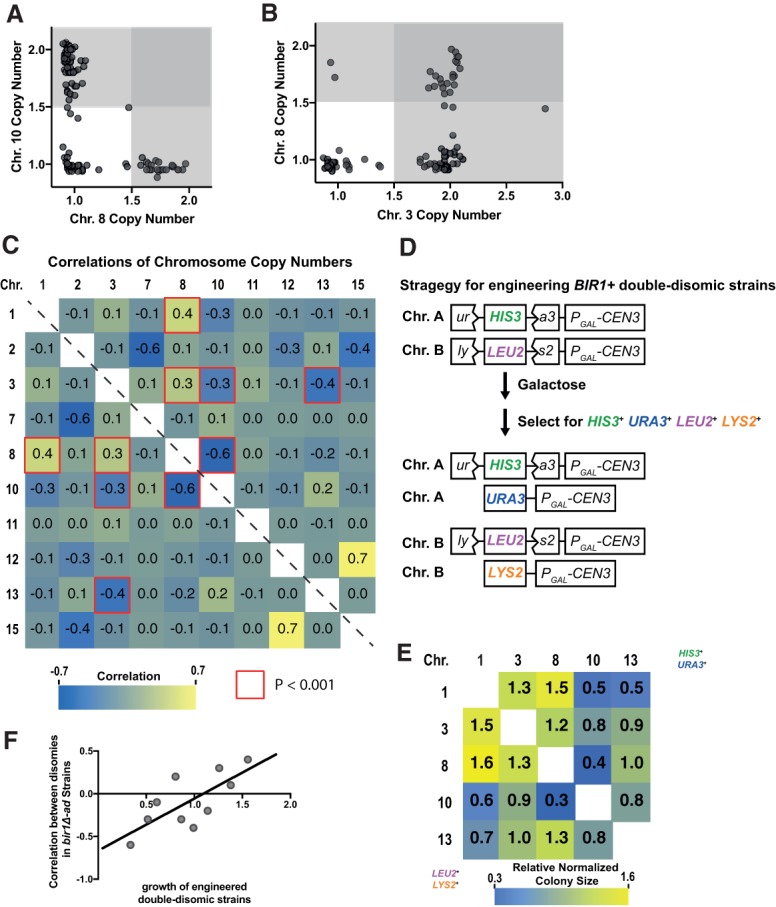
Genetic interactions between whole-chromosome disomies dictate patterns in complex aneuploidy. (*A*,*B*) Scatter plot of specific chromosome copy numbers of 102 *bir1Δ-ad* strains demonstrating positive (*B*) and negative (*A*) correlations between different chromosomal disomies. Copy number data are from read frequencies as in Figure 1C. The darker-gray regions contain strains that are aneuploid for both of the plotted chromosomes. (*C*) Heat map of the correlations between chromosome copy numbers in the 102 haploid *bir1Δ-ad* strains. Only correlations between chromosomes where aneuploidy was observed are shown. Each cell of the matrix contains the correlation coefficient between the two chromosomes written in the row and column. The red highlighted border represents correlations with *P* < 0.001 in the hypergeometric test. (*D*) Schematic of a system to engineered strains (*BIR1*^+^) that harbor two chromosome disomies. Strains were transformed with the construct *P_GAL_-CEN3 ura3::HIS3* on one chromosome (chromosome A) and *P_GAL_-CEN3 lys2::LEU2* on another chromosome (chromosome B). Chromosome nondisjunction was induced with galactose, and disomy of both chromosomes was selected for with all four auxotrophic markers (*URA3*, *HIS3*, *LYS2*, and *LEU2*). (*E*) Relative colony sizes of double-disomic strains on YPAD plates were normalized to the other values in their respective rows and columns. Colony sizes without normalization are shown in Supplemental Figure S2I. (*F*) Comparison of the correlation coefficients of the chromosome copy numbers in *bir1Δ-ad* strains (from *C*) and the relative fitness of engineered double-disomic strains (from *E*). Relative values are the average of the two values in *E* (column:row and row:column for each chromosome pair). Pearson's correlation coefficient = 0.73. Two-tailed *P*-value = 0.016.

If disomy of chromosome 10 gives an initial growth advantage, why does it not correlate with increased fitness in the adapted strains? Up to this point, we analyzed each chromosomal aneuploidy independently. However, 96% of the *bir1Δ-ad* strains had complex karyotypes, as defined by containing more than one chromosome copy number alteration (Fig. 1C). We next determined whether there were any correlations between the copy numbers of different chromosomes. The most striking pattern is a negative correlation between disomy of chromosomes 8 and 10. Seventy-six percent (78 of 102) of adapted strains have an elevated copy number of either chromosome 8 or 10, but only one strain has an increased copy number of both chromosomes (*P* = 2.6 × 10^−9^, hypergeometric test) (Fig. 3A). Conversely, a strong positive correlation is seen between chromosomes 8 and 3. Ninety-two percent of strains (24 of 26) with chromosome 8 gains have increases in chromosome 3 as well, while only 59% (45 of 76) of strains with a single copy of chromosome 8 have an extra copy of chromosome 3 (*P* = 0.001, hypergeometric test) (Fig. 3B). Comprehensive pairwise correlations between all aneuploid chromosomes in the *bir1Δ-ad* strains revealed five highly significant (*P* < 0.001) correlations (Fig. 3C). We conclude that aneuploidies of individual chromosomes do not act independently, which could help explain why aneuploidy of some chromosomes, such as chromosome 10, improved initial growth following *BIR1* deletion (Fig. 2B) but did not correlate with increased fitness in the adapted strains (Fig. 1E).

### Chromosome copy number correlations result from genetic interactions between aneuploid chromosomes

The strong anti-correlation between disomy of chromosomes 8 and 10 could result from functional redundancy in their adaptive advantage to *BIR1* deletion, resulting in a loss of positive selection for the second disomic chromosome. Alternatively, combination of both disomies could result in a synthetic negative genetic interaction independently of *BIR1* deletion. To test for genetic interactions between specific pairs of disomic chromosomes, we modified the aneuploidy induction system to engineer two chromosomal aneuploidies simultaneously (Fig. 3D). After induced missegregation via addition of galactose, disomy of both chromosomes was selected for on plates lacking histidine, uracil, leucine, and lysine. We engineered pairwise combinations of all five chromosomes that showed significant positive or negative copy number correlations in the *bir1Δ-ad* strains (Fig. 3E). The presence of an extra copy of both chromosomes was confirmed by quantitative PCR (qPCR). Colony sizes for each pair were measured and normalized to account for growth differences in individual aneuploidies (Fig. 3E; Supplemental Fig. S2I). Colony sizes ranged from 70% smaller to 60% larger than expected, demonstrating strong positive and negative genetic interactions between aneuploid chromosomes. These genetic interactions were not simply a result of increasing amounts of extra DNA, as there was no significant correlation between combined chromosome size and relative growth for the chromosome combinations that we tested (*P* = 0.48) (Supplemental Fig. S2J). We call these whole-chromosome-level genetic interactions chromosome copy number interactions (CCNIs).

To determine the degree to which CCNIs could explain the complex karyotype patterns observed in the *bir1Δ-ad* strains, we directly compared the colony sizes of the engineered double-disomic strains (Fig. 3E) with the chromosome copy number correlations in the *bir1Δ-ad* strains (Fig. 3C). The combination of disomies with the highest significant positive correlation in the *bir1Δ-ad* strains, chromosomes 1 and 8, had the largest relative colony sizes in engineered double-disomic strains. Similarly, the disomy pair with the highest significant negative correlation, chromosomes 8 and 10, had the smallest relative colony sizes (Fig. 3C,E,F; Supplemental Fig. S3A). Results were similar if the selection markers used for the two chromosomes were reversed (Fig. 3E). No negative interactions were seen with chromosome 2 disomy (Supplemental Fig. S2H,I). Overall, the correlation between growth of engineered disomic pairs and frequency of co-occurrence in *bir1Δ-ad* strains was significant (*r* = 0.73, *P* = 0.016) (Fig. 3F), demonstrating that aneuploidy patterns observed in complex karyotypes are directly affected by positive and negative genetic interactions between specific aneuploid chromosome pairs. This indicates that CCNIs play a key role in governing the formation of complex aneuploid karyotypes.

### The complexity of aneuploidy correlates with the severity of CIN induction

**Figure 4.**
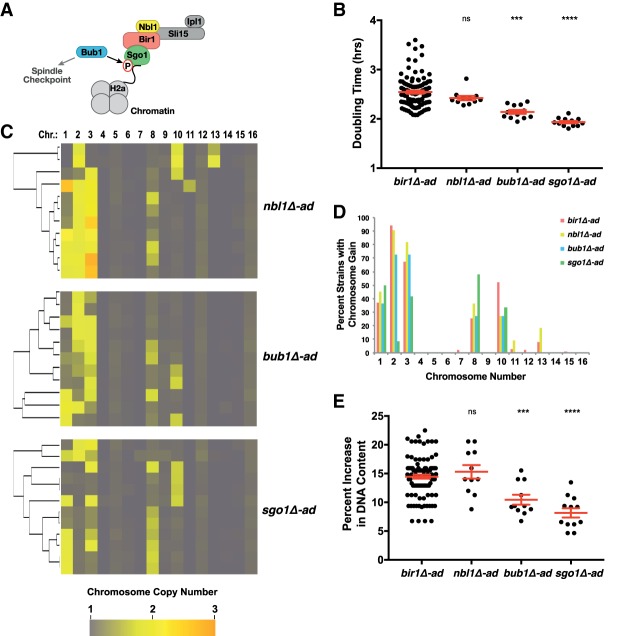
The degree of aneuploidy correlates with the severity of induced CIN. (*A*) Cartoon of the CPC and the regulators of its localization to chromatin. (*B*) Cell doubling times for each of the indicated strain types. (*C*) Heat map visualization of the clustered chromosome copy numbers of the *nbl1Δ-ad*, *bub1Δ-ad*, and *sgo1Δ-ad* strains. (*D*) Plot of the percentage of strains with a gain of a specific chromosome based on binarization of the data shown in *C* and Figure 1C. (*E*) Percentage change in overall DNA content as measured by taking the binarized copy number values of each chromosome, subtracting 1, multiplying each by the fraction of the genome represented by that chromosome, and then summing all of the chromosomes. Mean values and the standard errors are in red. (***) *P* ≤ 0.0001; (****) *P* < 0.00001.

We next tested how varying the level of induced CIN affects the resulting complex aneuploidy. To alter the amount of CIN induced, we used mutants that affect different aspects of the kinetochore–microtubule attachment error correction pathway (Fig. 4A). Deletions in *NBL1*, *BUB1*, and *SGO1* (Borealin, BUB1, and Shugoshin in humans) were adapted via clonal expansion in the same manner as the *BIR1* deletion strains. The growth rates of adapted strains demonstrate that *bir1Δ-ad* and *nbl1Δ-ad* have the strongest growth defects, followed by *bub1Δ-ad* strains, and the *sgo1Δ-ad* strains had the weakest phenotype (Fig. 4B). These results are consistent with previously published measurements of missegregation rates for mutants in *BIR1* and *SGO1* (Storchova et al. 2011; Campbell and Desai 2013). Whole-genome sequencing to determine the chromosome copy numbers for the adapted strains revealed very similar aneuploidy patterns for all four deletion mutants. The four mutants resulted in aneuploidy predominantly in the same five chromosomes (chromosomes 1, 2, 3, 8, and 10) (Fig. 4C,D). Additionally, the negative correlation between chromosome 8 and 10 disomy is also observed in these strains, although one *sgo1Δ-ad* strain did display disomy of both chromosomes (Fig. 4C). Although the general patterns of aneuploidy remained the same in the different adapted deletion strains, the degree of aneuploidy varied. *nbl1Δ-ad* and *bir1Δ-ad* had very similar increases in aneuploidy, whereas *bub1Δ-ad* and *sgo1Δ-ad* strains averaged comparatively less aneuploidy (Fig. 4E; Supplemental Fig. S3B). This trend correlates well with the degree of CIN observed in these strains. Together, these results suggest that increased karyotype complexity can result directly from elevated rates of chromosome missegregation.

### Ploidy greatly affects the patterns of chromosome copy number correlations

**Figure 5.**
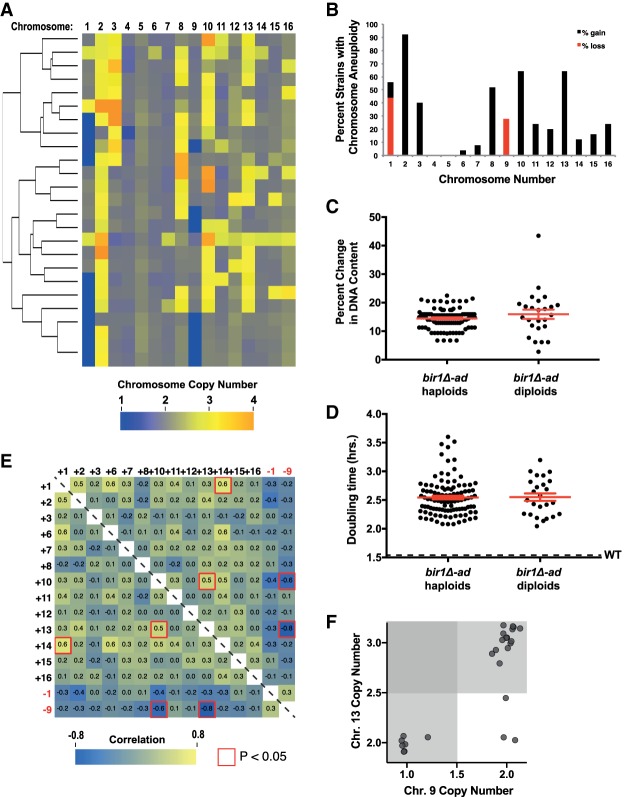
Ploidy greatly affects chromosome copy number correlations. (*A*) Heat map visualization of chromosome copy number values for diploid *bir1Δ-ad* strains clustered by similarity. Each of the 25 diploid *bir1Δ-ad* strains is represented as a row. (*B*) Frequency of the aneuploid chromosomes in the diploid *bir1Δ-ad* strains from binarization of the data shown in *A*. (*C*) Percentage change in overall DNA content as measured by taking the binarized copy number values of each chromosome, subtracting the basal ploidy 2, multiplying each by the fraction of the genome represented by that chromosome, and then summing the absolute values for all of the chromosomes. There was no significant difference between the two groups. *P* = 0.16. (*D*) Cell doubling times for the haploid and diploid *bir1Δ-ad* strains. There was no significant difference between the two groups. *P* = 0.91. (*E*) Heat map of the correlations between chromosome copy numbers for the 25 diploid *bir1Δ-ad* strains. Only correlations between chromosomes where aneuploidy was observed are shown. Each cell of the matrix contains the correlation coefficient between the two chromosomes written in the rows and columns. The red highlighted border represents correlations that had a *P* < 0.05 in the hypergeometric test. (*F*) Scatter plot of specific chromosome copy numbers of 25 diploid *bir1Δ-ad* strains demonstrating a negative correlation between monosomy of chromosome 9 and trisomy of chromosome 13. Copy number data are from read frequencies as in *E*. The darker-gray region contains strains that are aneuploid for both of the plotted chromosomes. Mean values and the standard error of each group are indicated in red with error bars.

We next sought to determine how changes in ploidy affect patterns in complex karyotypes. Compared with haploid genomes, diploids have a greater number of potential aneuploidy types to exploit for adaptation, such as chromosome loss (monosomy) and gain (trisomy) of only 50% more copies of a chromosome. In theory, this would provide more avenues to fine-tune adaptation through aneuploidy. To test this, we generated 25 diploid *bir1Δ* yeast strains, adapted them through clonal expansion, and subjected them to whole-genome sequencing (Fig. 5A). Similar to the adapted haploid strains, the diploids frequently accumulated extra copies of chromosomes 2, 3, 8, and 10 (Fig. 5B). In addition, the diploids had a much higher frequency of chromosome 13 gain (64% in diploids vs. 8% in haploids) (Figs. 1D, 5B). Although chromosomes 2, 3, 8, and 10 all had instances of tetrasomy, this was not the case for chromosome 13 (Fig. 5A). These data suggest that a 50% increase in chromosome 13 copy number provides a much better balance of growth benefit to fitness deficit when compared with a 100% increase in *bir1Δ-ad* strains. In addition to chromosome gains, losses of chromosomes 1 and 9 were also observed. Intriguingly, chromosome 1 was lost in some strains and gained in others, suggesting that the presence or absence of additional copies of this chromosome is largely inconsequential in adaptation to *BIR1* deletion. The percentage change of DNA content (normalized to the basal ploidy) for *bir1Δ-ad* strains was very similar for haploids and diploids (Fig. 5C), demonstrating that the aneuploidy burden that is tolerated by a cell scales with its ploidy. Curiously, there was no significant difference between the mean doubling times of *bir1Δ-ad* haploids and diploids despite the diploids having more avenues for adaptation (Fig. 5D). Overall, these data show that initial ploidy is an important determinant in complex aneuploidy patterns.

We next determined the pairwise correlations between specific chromosome aneuploidy types in diploid *bir1Δ-ad* strains (Fig. 5E). Surprisingly, the most significant negative correlation in haploids, between gain of chromosomes 8 and 10, was not present in the diploids. Instead, the most significant correlations are between the loss of chromosome 9 and the gain of chromosome 10 or 13 (*P* = 0.003 and *P* = 7 × 10^−5^, respectively). Although 92% (23 out of 25) of strains had either chromosome 13 gain or chromosome 9 loss, none of them had both (Fig. 5E,F). As with chromosomes 8 and 10 in haploids, the anti-correlation between chromosome 13 trisomy and chromosome 9 monosomy is associated with a negative CCNI. The growth of cells engineered with both trisomy of chromosome 13 and monosomy of chromosome 9 was approximately half the size of those with chromosome 9 monosomy alone (*P* < 0.0001, Supplemental Fig. S3C). This demonstrates that CCNIs also play a role in shaping complex karyotypes with a diploid base ploidy. We conclude that chromosome copy number correlations and CCNIs are observed in multiple ploidy states, but the patterns change dramatically.

### Chromosome copy number correlations are seen in cancer karyotypes

**Figure 6.**
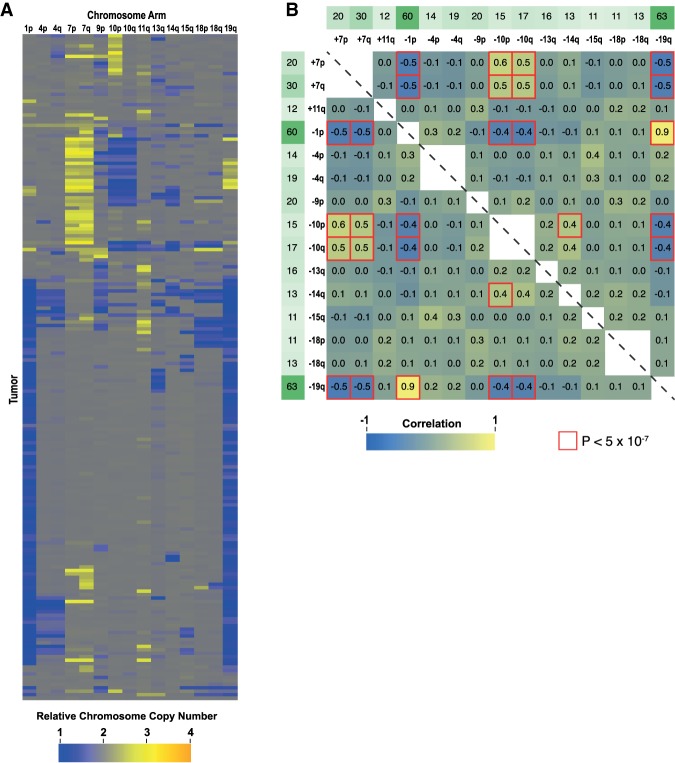
Identification of chromosome copy number correlations in brain LGG tumors. (*A*) Visualization of the chromosome arm karyotypes from brain LGG tumor samples. Cancer karyotype data was acquired from the CGH data from the TCGA database. Each of the 193 LGG karyotypes is represented as a row, with the column indicating the chromosome copy number of chromosome arms. Only chromosome arms where aneuploidy was present in >10% of the tumor samples are shown. (*B*) Pairwise correlation coefficients for chromosome arm aneuploidy are shown as a heat map. The frequency of each aberration is shown in green as a percentage. The red highlighted border indicates *P* < 5 × 10^−7^ in the hypergeometric test. See Supplemental Figure S4 for similar analysis of four additional tumor types.

To determine whether cancer karyotypes have patterns similar to those that we observed in adapted CIN yeast strains, we analyzed competitive genome hybridization (CGH) data from The Cancer Genome Atlas (TCGA) database. Cancer karyotypes for 15 different cancer types were analyzed with a matrix of pairwise correlations between different aneuploidies. The cancers with the five highest percentages of predominantly whole-chromosome or whole-arm complex aneuploidy were analyzed in higher detail (Fig. 6A; Supplemental Fig. S4; Supplemental Table S2). Strong correlations were observed in all five cancer types (Fig. 6A; Supplemental Fig. S4). Correlation patterns in lower-grade glioma (LGG) stood out as being especially similar to the *bir1Δ-ad* strains, with both strong positive and negative correlations for a subset of chromosomes (Fig. 6A,B). LGG karyotypes largely fell into two categories: those with (1) loss of 1p and 19q or (2) gain of chromosome 7 and loss of chromosome 10 (Fig. 6B). The combined loss of 1p and 19q is the result of a frequent translocation between those two chromosomes. Intriguingly, strains that harbor this translocation almost never have a gain of chromosome 7 or loss of chromosome 10 (Fig. 6B). Other common chromosome aberrations such as loss of chromosome 18 did not have any strong correlations, suggesting specificity in the negative correlation between the two main karyotype classes. We conclude that chromosome copy number correlations are also present in cancer karyotypes and may reflect genetic interactions between different aneuploid chromosomes (CCNIs).

### Additional competitive adaptation of *bir1Δ-ad* strains leads to convergent optimized complex karyotypes

**Figure 7.**
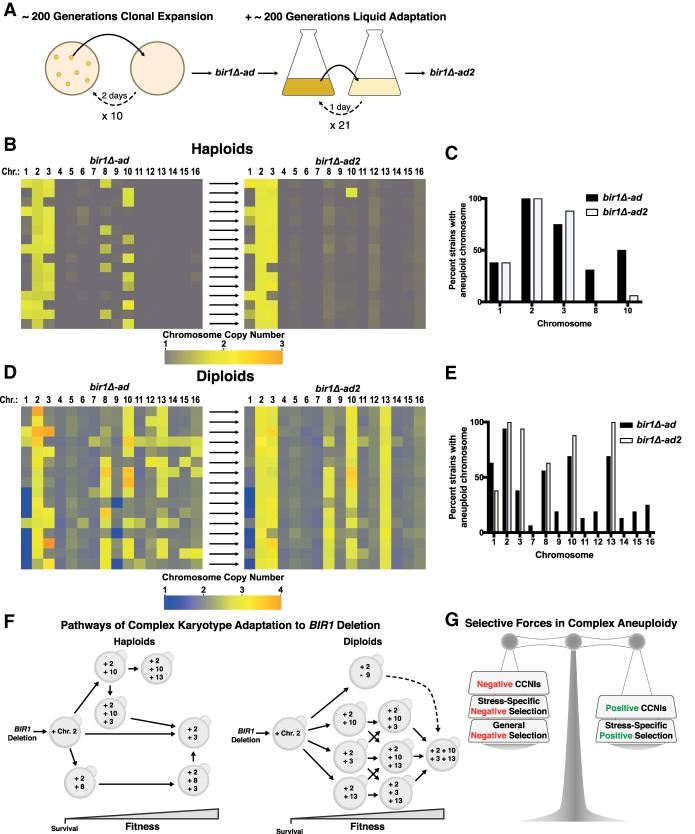
Additional adaptation leads to convergence on “ideal” karyotypes in *BIR1* deletion strains. (*A*) Graphical representation of the adaptation process on solid (see Fig. 1A) followed by liquid medium. On solid medium, the *bir1Δ* strains were struck out for single colonies and allowed to grow for 2 d at 30°C. After 10 rounds of growth on solid medium, a subset of *bir1Δ-ad* strains, selected to represent a diverse set of karyotypes, was grown for an additional ∼200 generations in liquid medium. Liquid cultures were diluted each day for 21 d, generating further adapted haploid/diploid *bir1Δ-ad2* strains. (*B*) Heat map visualization of chromosome copy number values for the haploid *bir1Δ* strains before (see Fig. 1C) and after adaption in liquid medium. The *bir1Δ-ad* strains at the *left* directly correspond to the *bir1Δ-ad2* strains at the *right*. (*C*) Frequency of aneuploidy for each chromosome in the haploid *bir1Δ-ad* and *bir1Δ-ad2* strains based on binarization of the data in *B*. (*D*) Heat map visualization of chromosome copy number values for the diploid *bir1Δ* strains before (see Fig. 5A) and after adaption in liquid medium. The *bir1Δ-ad* strains at the *left* directly correspond to the *bir1Δ-ad2* strains at the *right*. (*E*) Frequency of aneuploidy for each chromosome in the diploid *bir1Δ-ad* and *bir1Δ-ad2* strains based on binarization of the data in *D*. (*F*) An empirical model depicting the pathways of adaptation to *BIR1* deletion based on synthesizing data from Figures 1, 2, 3, and 5 and this figure. Adaptation starts with the gain of chromosome 2, which appears to be essential for initial survival. Following chromosome 2 gain, many different possible paths can be taken by haploid and diploid yeast during the formation of complex karyotypes. Note that some chromosome aneuploidies (disomy of chromosome 10 in haploids and monosomy of chromosome 9 in diploids) provide a growth advantage early in adaptation but must become euploid again to achieve the “optimal” complex karyotype. (*G*) Illustration of the selective forces that determine whether the aneuploidy of a chromosome is advantageous.

Given that certain patterns in the karyotypes of *bir1Δ-ad* strains had strong correlations with increased growth rates, it is somewhat surprising that not all of the strains adapted to have the most advantageous patterns. We reasoned that perhaps if the adapted *bir1Δ* strains were given more time to adapt in a more competitive environment, they might converge on an “ideal” karyotype. Alternatively, further adaptation could allow the strains to find alternate ways to independently obtain the advantages conferred by the aneuploidy and then eliminate the disadvantages by returning to the euploid state (Yona et al. 2012). We therefore selected 16 haploid and 16 diploid strains for further adaptation for an additional ∼200 generations in rich liquid medium (Fig. 7A). We refer to these further adapted strains as *bir1Δ-ad2*. The adaptation in liquid medium did not greatly affect the CIN phenotype of the further adapted strains, as the 16 *bir1Δ-ad2* diploids had little to no change in benomyl sensitivity when compared with the *bir1Δ-ad* strains (Supplemental Fig. S5A). In the haploid *bir1Δ-ad2* strains, aneuploidy of chromosomes 8 and 10 was almost completely lost (Fig. 7B,C). This resulted in strains with more similar karyotypes, as the pooled standard deviation decreased from 0.18 to 0.13 after liquid adaptation. In contrast, levels for chromosomes 1 and 2 were largely unchanged. Notably, all four strains that started with chromosome 10 disomy and chromosome 3 monosomy lost a copy of chromosome 10 and gained a copy of chromosome 3. This suggests that, in haploids, the “optimal” *bir1Δ* karyotype is disomy of chromosomes 2 and 3, with chromosome 10 disomy being excluded due to the negative CCNI between aneuploidy of chromosomes 3 and 10 (Fig. 3C,E). Interestingly, the diploid *bir1Δ-ad2* strains became much more homogenous in their karyotypes, with a decrease in pooled standard deviation from 0.36 to 0.17 after liquid adaptation. Trisomy of chromosomes 2, 3, 10, and 13 is now present in nearly all of the further adapted strains (Fig. 7D,E). Strikingly, all three *bir1Δ-ad* strains that started off with chromosome 9 monosomy regained a copy of chromosome 9 and acquired an extra copy of chromosome 13. Both of these changes occurred within 1 wk of each other in all three strains and occurred within the first 2 wk of liquid adaptation (Supplemental Fig. S5B). We conclude that certain types of aneuploidy that give an initial growth advantage, such as gain of chromosome 10 in haploids and loss of chromosome 9 in diploids, will eventually be lost due to the comparatively better fitness increases of other, incompatible types of aneuploidy (chromosome 3 gain in haploids and chromosome 13 gain in diploids). Together, these results suggest that although there are often many different, sometimes conflicting paths to obtaining an ideal karyotype, cell populations with high levels of CIN will eventually converge on a common complement of aneuploid chromosomes.

## Discussion

In this study, we developed a system for studying how complex karyotypes arise from extremely high rates of CIN. Mutations in the kinetochore–microtubule error correction pathway provide an increase in the frequency of aneuploidy while simultaneously creating the selective pressure to adapt via aneuploidy. We identified strong correlations and anti-correlations between specific aneuploid chromosome pairs in the resulting complex karyotypes and hypothesized that these patterns result from genetic interactions between certain aneuploid chromosomes. We then identified genetic interactions between whole-chromosome copy number alterations (CCNIs) in engineered double-aneuploid strains that match the patterns observed in the adapted CIN strains. After additional competitive adaptation of the CIN strains, we observed an increase in homogeneity of the strains as they converged on an optimal karyotype.

We present a model of the different paths that yeast take in order to get to a final ideal aneuploid state that minimizes the growth defects resulting from *BIR1* deletion (Fig. 7F). These paths of adaptation were based on (1) comparing cellular fitness with the different aneuploid states in the adapted strains (Figs. 1E, 2B), (2) comparing the karyotypes before and after liquid adaptation (Fig. 7B–E), and (3) the CCNIs observed between aneuploid chromosomes (Fig. 3E; Supplemental Fig. S3A,C). While the paths of adaptation are quite different for haploid and diploid cells, they have several things in common. For both ploidies, there are stepwise increases in aneuploidy and corresponding increases in fitness. The paths always start with the gain of chromosome 2, which is required for initial survival. After that, the cultures can acquire a number of different aneuploidies that all provide some selective advantage. These aneuploidies will sometimes lead down a direct route that quickly leads to the final optimal karyotype, such as the gain of chromosome 3 in haploids. Alternatively, the cultures will instead take a detour via an aneuploidy that is initially advantageous but, paradoxically, also provides a temporary barrier to obtaining the ideal complement of aneuploid chromosomes. Examples of this include gain of chromosome 10 in haploids or loss of chromosome 9 in diploids. These aneuploidies are eventually replaced due to negative CCNIs with copy number alterations that are even more advantageous. Our results demonstrate that aneuploidy can provide a surprisingly versatile mechanism of adaptation, with many different chromosomal aneuploidies able to provide an initial competitive advantage under selective conditions. Despite these divergent starting points, chromosomally unstable cell populations settle on an ideal complex karyotype through the gain and loss of chromosomes over time.

Synthetic genetic interactions between chromosomes have been reported before, as disomy of chromosome 6 is tolerated only in conjunction with a concomitant gain of chromosome 13. This interaction is due to imbalanced expression of the *TUB2* gene on chromosome 6 and can be compensated for by increased expression of *TUB1* from chromosome 13 (Torres et al. 2007; Anders et al. 2009). Here we demonstrate that both positive and negative whole-chromosome genetic interactions are potentially quite common and have strong effects on shaping complex aneuploid karyotypes. As of yet, we do not know the basis behind these genetic interactions and whether they typically result primarily from genetic interactions between pairs of genes, as with chromosomes 6 and 13, or due to cumulative effects of many genes, as has been reported for the growth defects that result from aneuploidy in general (Bonney et al. 2015). However, the specificity of the CCNIs among different chromosomes would suggest that they are likely the result of imbalances in a few specific genes. Another open question is whether cells frequently adapt to maintain the beneficial aspects of aneuploidy while finding ways to minimize the negative effects of genetic interactions between chromosomes.

Whether aneuploidy of a particular chromosome is advantageous depends on the balance between positive and negative selective forces (Fig. 7G). The key positive selective pressure for aneuploidy is the increased expression of genes on a chromosome that specifically relate to the selective forces acting on the cell population (Hughes et al. 2000; Rancati et al. 2008; Ryu et al. 2016). Negative selective pressure can come from the aberrant expression of many different genes simultaneously as well as stress-specific negative selection (Bonney et al. 2015; Chen et al. 2015). Here, we show that specific genetic interactions between aneuploid chromosomes can contribute either positively or negatively to the selective forces on aneuploidy. Additionally, the selective advantage for aneuploidy is heavily influenced by the original ploidy of the cells, as haploid and diploid yeast display largely divergent aneuploidy patterns. Furthermore, we found that the degree of aneuploidy resulting from CIN in diploids is, on average, doubled in comparison with haploids. These results are in agreement with the observation that gaining an extra chromosome in diploids causes much weaker phenotypes than gaining an extra chromosome in haploids (Beach et al. 2017). On the extreme end, tetraploidy in yeast has been shown to greatly encourage adaptation via aneuploidy (Selmecki et al. 2015). Together, these results highlight how subtle changes in the forces that select for and against aneuploidy can have a strong impact on the aneuploidy landscape in a cell population.

In cancer cell lines, higher amounts of aneuploidy are directly correlated with increased CIN rates, as measured by the frequency of lagging chromosomes (Duesberg et al. 1998; Nicholson and Cimini 2013). Since engineered aneuploid cells often display CIN (Sheltzer et al. 2011; Zhu et al. 2012; Passerini et al. 2016), this correlation has often been interpreted as aneuploidy being upstream of CIN (Nicholson and Cimini 2013). Here we show that the reciprocal relationship also exists. Yeast mutants with higher rates of CIN adapt to have proportionally more aneuploidy following adaptation. This may also explain why we observed much more complex karyotypes than previous studies that induced lower amounts of CIN and examined the resulting aneuploidy (Chen et al. 2012).

By analyzing the karyotypes of many different tumor cell populations, we identified that certain cancer types have strong correlations between specific somatic copy number alterations (SCNAs). A previous pan-cancer analysis of complex karyotypes revealed many positive correlations between whole-chromosome copy number changes (Ozery-Flato et al. 2011). Here, we found that restricting the analysis to certain cancer types allows for SCNAs at frequencies high enough to identify strong negative correlations in addition to positive correlations. These correlations are quite frequent and are suggestive of chromosome-level synthetic genetic interactions. Correlations between different aneuploidies in cancer identified in this manner could provide a starting point for identifying CCNIs in human cells. Additionally, our discovery that multiple distinct paths can lead to refined adaptive karyotypes provide a first glimpse into the paths of complex karyotype formation in cancer.

## Materials and methods

### Yeast strains and media

All yeast strains and plasmids used in this study are listed in Supplemental Table S1. Strains were grown in yeast extract/peptone supplemented with 40 µg/mL adenine-HCl (YPA) and 2% sugars. Benomyl (Sigma-Aldrich, 381586) and 5-FOA (Chempur, 220141-70-8) were used at concentrations of 10 µg/mL and 1 mg/mL, respectively. Cultures were incubated at 30°C. Genetic manipulations such as gene deletions were carried out as described (Longtine et al. 1998). Haploid strains with deletions of CIN genes were generated via tetrad dissection of heterozygous diploids. Homozygous diploid *BIR1* deletion mutants were made one of two ways. Two heterozygous diploids were deleted of either the MATa or MATα locus to allow for mating between diploids. The resulting tetraploid strains were then sporulated, and tetrads were dissected. Alternatively, both copies of the endogenous *BIR1* locus in a diploid strain were deleted in the presence of *BIR1* linked to *URA3* on a minichromosome. The minichromosome was then selected against using 5-FOA. Single disomic strains (N + 1 aneuploid strains) were constructed using a conditional centromere as described in Anders et al. (2009). In this system, the function of the centromere is repressed by the galactose-inducible promoter, causing frequent chromosome missegregation. Endogenous centromeres were targeted using recombination sites both upstream of and downstream from the centromere and subsequently replaced by the galactose-repressable centromere P_GAL1_-CEN3 and either *URA3* or *LYS2*. This insert was then disrupted by insertion of plasmids with pieces of *URA3* or *LYS2* linearized with the Stu2 or EcoRV restriction enzymes, respectively, and selected for with *HIS3* or *LEU2* genes also present on the plasmid. Haploid strains containing P_GAL1_-CEN3 *ura3*::*HIS3* and/or P_GAL1_-*CEN3 lys2*::*LEU2* constructs were grown to log phase in YPA plus 2% dextrose (YPAD) and transferred to YPA plus 1% galactose and 1% raffinose (YPAGR) medium for 3 h. The cells were subsequently plated onto selection plates with complete synthetic medium (CSM) lacking either uracil and histidine or lysine and leucine. The resulting aneuploid strains were verified by qPCR. For double-disomic strains (N + 2 aneuploid strains), P_GAL1_-CEN3 *ura3*::*HIS3* and P_GAL1_-*CEN3 lys2*::*LEU2* were inserted on separate chromosomes. After induction for 3 h in YPAGR, the cultures were first plated on CSM lacking uracil and histidine. Single colonies were then selected on CSM plates lacking lysine and leucine. For engineering simultaneous loss and gain of chromosomes in diploid strains, P_GAL1_-CEN3 *URA3* and P_GAL1_-*CEN3 lys2*::*LEU2* were inserted into separate chromosomes to be lost and gained, respectively. The cells were induced as stated above and selected first on plates containing 5-FOA. Subsequently, single colonies were grown on CSM plates lacking lysine and leucine. The respective aneuploidies of the selected strains were verified using qPCR.

### Adaptation through clonal expansion

Fresh *BIR1*-deleted haploids were obtained by tetrad dissection of heterozygous diploids on YPAD. After 4 d of growth, small colonies (*bir1Δ*) from tetrads with two large colonies (*BIR1*^+^) were streaked out for singles on fresh YPAD plates. Six colonies from each initial colony were then selected for further analysis by clonal expansion. Every 2 d, one large colony from each strain was streaked out on a fresh plate. After 10 clonal expansions, the strains were kept frozen at −80°C in 25% glycerol. Subsequent experiments were performed from these frozen stocks. The *bir1Δ* genotype was confirmed by hygromycin resistance as well as genome sequencing of the adapted strains.

### Doubling time measurements

Overnight cultures were diluted to an optical density (OD) at 600 nm of 0.01 in YPAD. OD measurements were taken 3, 4, 5.5, 7, and 8.5 h after induction. The measurements were fit to logarithmic curves using Microsoft Excel to calculate doubling times.

### Colony size measurements

Yeast strains were streaked for single colonies on YPAD plates and incubated for 40 h. The plates were imaged using an SPimager (S&P Robotics, Inc.) fitted with a Canon Rebel XSi dSLR camera, and the images were analyzed using ImageJ. After thresholding, colony sizes were measured using the “analyze particles” function with the following settings: particle size: 0.03–3 mm^2^; circularity: 0.90–1. Median colony sizes from multiple plates were averaged for each strain.

### Flow cytometry

Log-phase cultures (OD_600_ ∼ 1.0) were pelleted and resuspended in 50 mM sodium citrate and sonicated to disperse cell clumps. Subsequently, the cells were treated with 250 µg/mL RNase A (Sigma-Aldrich, R6513) and 1 mg/mL Proteinase K (Sigma-Aldrich, P2308) overnight at 37°C. Finally, the cells were resuspended in 50 mM sodium citrate solution containing 1 µM SYTOX green (Thermo Fisher, 10768273). Samples were run on BD FACSCallibur flow cytometer equipped with a 15-mW 488-nm laser. Maximum count peaks for fluorescence intensities were calculated using the BD FACSDiva 8.0.1 software.

### Next-generation sequencing and data analysis

DNA from saturated overnight cultures was isolated using the Wizard genomic DNA purification kit (Promega). The samples were then fragmented to ∼500 base pairs (bp) using the Bioruptor Pico sonicator for two to three cycles (30 sec on/off). The samples were subsequently run on a 0.8% agarose gel to verify the fragment length. DNA libraries were prepared using the NEBNext Ultra II DNA library preparation kit for Illumina (New England Biosciences). AMPure XP beads were used for size selection. Twelve to 16 strains per run were multiplexed with NEBNext Multiplex oligos (96 index primers) and mixed at equimolar ratios. The multiplexed samples were sequenced using the 50-bp paired-end setting on an Illumina HiSeq 2500 system at the Vienna Biocenter Next-Generation Sequencing Facility (VBCF). The demultiplexed data sets were then aligned to the yeast genome using Bowtie2 (version 2.2.9; http://bowtie-bio.sourceforge.net/bowtie2) and converted to bed files using SAMtools (version 1.3.1; http://samtools.sourceforge.net) and Bedtools (version 2.14, http://bedtools.readthedocs.io). The resulting bed files were used to calculate chromosome copy numbers for read densities using custom-made Python2 scripts. To normalize for differences in chromosome sizes, only the 15 kb closest to the telomeres were used. The value for the second-lowest quartile chromosomes was used for normalization. Mutations in the *bir1Δ-ad* strains were identified by taking the output from Bowtie2 and running mpileup function in SAMtools. The data were filtered by quality score and read depth. Next, BCFtools (version1.3.1) was implemented to convert the bcf files generated by mpileup to variant call format (.vcf) files. Subsequently, VCFtools (version0.1.13) was used to compare all mutations found in our test strains with mutations already identified in the diploid parent strain. Last, a custom-made Python script generated lists containing the strain identity, the coordinates of the mutation (in base pairs), and the type of mutation (coding/noncoding). We generated a list of CIN genes in the yeast genome from the *Saccharomyces* Genome Database associated with six specific GO terms: colony sectoring: increased; chromosome segregation: abnormal; chromosome/plasmid maintenance: decreased rate; chromosome/plasmid maintenance: abnormal; chromosome segregation: premature; and chromosome segregation: decreased. GO term enrichment tests were conducted using the Panther Classification System Web site (http://pantherdb.org) with the settings model organism: “*S. cerevisiae*”; “statistical overrepresentation test”; and “GO biological process complete.”

### Microscopy

Strains were grown overnight, subsequently diluted 100-fold, and grown for 5 h to mid-log phase. Cells were pelleted by brief centrifugation, washed, and resuspended in 100 mM phosphate buffer (pH 7.4). They were then placed on 1% agarose pads supplemented with complete synthetic medium, covered with a coverslip, and sealed around the edges with VALAP (a 1:1:1 mixture of petroleum jelly [Vaseline], lanolin, and paraffin [Thermo Fisher Scientific] by weight). Time-lapse imaging was performed on an Olympus cellSens microscopy system (Olympus Corporation) fitted with an Olympus cellVivo system for temperature control at 30°C. A 60× 1.42 NA oil immersion Olympus plan apochromat objective and an ORCA-Flash4.0 V2 sCMOS camera (Hamamatsu) were used for imaging. *Z*-sections were taken with 11 0.7-µm steps with Olympus cellSens version 1.18 software (Olympus Corporation). Images were collected every 15 min for a period of 4 h. Image analyses, including maximum intensity projections and contrast adjustments, were performed using ImageJ. The images shown were collected on the same day, and contrast was adjusted identically. For quantification of the missegregation rates the GFP-labeled chromosome 4, foci were followed over time through chromosome segregation. When both foci ended up in either the mother or daughter cell after complete nuclear division (judged by the background nuclear fluorescence), the division was scored as an event of missegregation. The percentage of all missegregation events in a strain out of the total number of nuclear divisions is reported as the missegregation rate.

### qPCR

Small amounts of cells from plates were lysed in 0.02 N NaOH for 10 min at 100°C in a thermocyler. The lysates were then centrifuged to pellet cellular debris, and the supernatants were collected. Each 20-µL reaction contained 10 µL of GoTaq qPCR master mix (Promega), 1 µL of lysate, and 1 µM each primer. Primers were in noncoding regions on each arm of the chromosome. The reactions were set up in 96-well plates (Eppendorf twin.tec real-time PCR 96-well plate) and cycled using a Mastercycler RealPlex^2^ (Eppendorf). Cycling conditions were for 5 min at 95°C followed by 40 cycles of 15 sec at 95°C and 1 min at 60°C. Dissociation curves were performed to verify that no secondary products had been amplified. C_t_ values were determined using the automatic thresholding of the Eppendorf RealPlex^2^ software. All reactions were run in duplicate along with the appropriate wild-type or parental controls. Chromosome copy numbers were calculated using a slightly modified ΔΔC_t_ method (Schmittgen 2001). The C_t_ values from duplicates were averaged and used to obtain the ΔC_t_, which was subsequently raised to the negative power of 2 to give the fold change. The ratio of fold change of the test strains to that of a wild-type strain was calculated to obtain the values for the chromosome copy numbers.

### Cancer genome databases and data analysis

Copy number variation (CNV) files were downloaded from the Genomic Data Commons (GDC) data portal (https://portal.gdc.cancer.gov) on October 19, 2017. Relative copy numbers were determined from each chromosome arm using a custom Python2 script. Tumor samples containing chromosome arms with large insertions or deletions (mean and median copy numbers differed by >0.2) were excluded from analysis. Karyotypes with no complex aneuploidy (fewer than two copy number aberrations) were not included in the statistical analyses.

### Liquid adaptation

Overnight saturated cultures were first diluted to OD_600_ of 0.1. Subsequently, the cultures were diluted 1000-fold into 200 µL of YPAD in 96-well plates (Nunc 96 deep-well plates: 2-mL volume) and covered with a Breathe-Easier strip (Sigma, 2763624). These plates were fixed onto an incubator (New Brunswick Innova 4000 benchtop incubator shaker) using custom-made holders and incubated with shaking at 300 revolutions per minute for 24 h at 30°C. Each day, the cultures were diluted 1000-fold into fresh medium.

### Statistics

Unpaired *t*-tests were performed in Prism 7 (Graphpad). Hypergeometric tests were performed in Python2 using the “hypergeom.pmf” function in the scipy.stats module. Pearson correlation coefficients were calculated using either the “pearsonr” function in the scipy.stats module in Python2 or the “correl” function in Microsoft Excel.

## Supplementary Material

Supplemental Material
