# Supplementary material for: Genetic interactions between specific chromosome copy number alterations dictate complex aneuploidy patterns
Source: Genes Dev. 2018 Dec 1;32(23-24):1485–98. doi: 10.1101/gad.319400.118 (PMC6295164; doi:10.1101/gad.319400.118)
Supplement: Supplemental Material [file supp_32_23-24_1485__index.html]

Genetic interactions between specific chromosome copy number alterations dictate complex aneuploidy patterns — Supplemental Material 

# Genetic interactions between specific chromosome copy number alterations dictate complex aneuploidy patterns

## Supplemental Material

- Supplemental\_Table\_S2.pdf
- Supplemental\_Table\_S3.pdf
- Supplemental\_Table\_S4.pdf
- Supplemental\_Figure\_S1.pdf
- Supplemental\_Figure\_S5.pdf
- Supplemental\_Figure\_S2.pdf
- Supplemental\_Figure\_S4.pdf
- Supplemental\_Figure\_S3.pdf
- Supplemental\_Table\_S1.pdf
