# Supplementary material for: Genetic interactions between specific chromosome copy number alterations dictate complex aneuploidy patterns
Source: Genes Dev. 2018 Dec 1;32(23-24):1485–98. doi: 10.1101/gad.319400.118 (PMC6295164; doi:10.1101/gad.319400.118)
Supplement: Supplemental Material [file supp_gad.319400.118_Supplemental_Table_S2.pdf]

Table S2. Filtering of cancer karyotypes based on type of aneuploidy. Related to Figure 6.

Cancer type	Number of samples	Samples with only whole chromosome/ arm aneuploidy	Number with complex whole chromosome/arm aneuploidy	Percent with complex whole chromosome/ arm aneuploidy
Breast Invasive Carcinoma	1096	214	177	16
Glioblastoma Multiforme	593	170	161	27
Ovarian Serous Cystadenocarcinoma	573	13	11	2
Lung Adenocarcinoma	518	114	97	19
Uterine Corpus Endometrial Carcinoma	547	323	137	25
Kidney Renal Clear Cell Carcinoma	532	273	253	48
Head and Neck Squamous Cell Carcinoma	531	120	98	18
Brain Lower Grade Glioma	514	225	193	38
Thyroid Carcinoma	505	468	42	8
Lung Squamous Cell Carcinoma	504	59	49	10
Prostate Adenocarcinoma	498	198	75	15
Skin Cutaneous Melanoma	470	91	78	17
Colon Adenocarcinoma	458	198	138	30
Stomach Adenocarcinoma	443	159	119	27
Bladder Urothelial Carcinoma	412	65	47	11
