# Supplementary material for: Genetic interactions between specific chromosome copy number alterations dictate complex aneuploidy patterns
Source: Genes Dev. 2018 Dec 1;32(23-24):1485–98. doi: 10.1101/gad.319400.118 (PMC6295164; doi:10.1101/gad.319400.118)
Supplement: Supplemental Material [file supp_gad.319400.118_Supplemental_Table_S3.pdf]

Table S3. List of genes mutated in the *bir1Δ-ad* strains.

Systematic	Standard	Mutant	Residue	CIN gene
tS(UGA)I	SUP17	missense	A18G	no
tV(AAC)G2	None	nonsense	E15*	no
YAL051W	OAF1	missense	V939I	no
YAR028W	None	missense	D32N	no
YCR017C	CWH43	missense	E250G	no
YCR021C	HSP30	missense	R11C	no
YCR033W	SNT1	missense	F870I	yes
YDL101C	DUN1	missense	A307V	yes
YDL137W	ARF2	missense	S147C	no
YDL145C	COP1	missense	I307S	no
YDL156W	CMR1	missense	L27F	yes
YDL171C	GLT1	missense	D1583V	no
YDL190C	UFD2	missense	Q88E	no
YDL243C	AAD4	indel	70	no
YDR104C	SPO71	missense	D814N	no
YDR142C	PEX7	missense	L253I	no
YDR159W	SAC3	missense	I1097L	no
YDR180W	SCC2	missense	N63Y	yes
YDR189W	SLY1	missense	D151N	yes
YDR190C	RVB1	missense	T322K	no
YDR238C	SEC26	missense	F555L	no
YDR302W	GPI11	missense	R29L	no
YDR304C	CPR5	missense	V188L	no
YDR321W	ASP1	missense	R55I	no
YDR325W	YCG1	missense	G677R	yes
YDR333C	RQC1	indel	565	no
YDR335W	MSN5	missense	H426Y	no
YDR387C	CIN10	missense	S311T	yes
YDR389W	SAC7	missense	L511F	no
YDR422C	SIP1	missense	E357D	no
YDR440W	DOT1	missense	H142N	no
YDR451C	YHP1	missense	A273T	no
YDR492W	IZH1	missense	E35K	no
YDR523C	SPS1	missense	F248S	no
YDR527W	RBA50	missense	G22V	no
YEL060C	PRB1	missense	G304R	no
YER002W	NOP16	missense	D122V	no
YER008C	SEC3	missense	Q495R	no
YER027C	GAL83	missense	P78S	no
YER043C	SAH1	missense	R241C	no
YER053C	PIC2	missense	N49Y	no
YER066W	RRT13	missense	G93S	no
YER095W	RAD51	missense	G40V	yes
YER109C	FLO8	missense	A529T	no
YER116C	SLX8	missense	P150S	yes
YER154W	OXA1	missense	D239H	no
YER155C	BEM2	missense	H2114Q	no

YER167W	BCK2	missense	S414P	no
YER172C	BRR2	missense	K351N	no
YER172C	BRR2	missense	N352Y	no
YER173W	RAD24	missense	T622A	yes
YER176W	ECM32	indel	781	no
YFL002C	SPB4	missense	R207S	no
YFL013C	IES1	missense	D309V	no
YFL013C	IES1	nonsense	Y572*	no
YFL021W	GAT1	missense	H182Y	no
YFL024C	EPL1	indel	Q790	no
YFL024C	EPL1	indel	Q790	no
YFR015C	GSY1	missense	A639T	no
YFR029W	PTR3	missense	D19E	no
YFR040W	SAP155	missense	D780H	no
YGL017W	ATE1	missense	A274S	no
YGL203C	KEX1	missense	P43S	no
YGL206C	CHC1	missense	P1463Q	no
YGL207W	SPT16	missense	W54R	yes
YGL207W	SPT16	missense	T713A	yes
YGR054W	None	missense	P242Q	no
YGR070W	ROM1	missense	V248A	no
YGR080W	TWF1	missense	E51D	no
YGR090W	UTP22	missense	S819L	no
YGR130C	None	indel	190	no
YGR142W	BTN2	indel	310	no
YGR208W	SER2	missense	A271T	no
YGR241C	YAP1802	indel	514	no
YGR253C	PUP2	missense	D71N	yes
YGR257C	MTM1	indel	344	no
YGR271C-A	EFG1	missense	L118F	no
YGR271W	SLH1	missense	N924K	no
YHL008C	None	indel	449	no
YHL034C	SBP1	missense	V44I	no
YHL041W	None	missense	S101F	no
YHR030C	SLT2	missense	L159F	no
YHR042W	NCP1	missense	L315M	no
YHR046C	INM1	missense	G112V	no
YHR072W	ERG7	missense	S612F	no
YHR078W	None	missense	C408Y	no
YHR106W	TRR2	missense	C165F	no
YHR117W	TOM71	missense	T293A	no
YHR138C	None	missense	V27A	no
YIL010W	DOT5	missense	S14F	yes
YIL042C	PKP1	missense	R97K	no
YIL073C	SPO22	nonsense	Q38*	no
YIL078W	THS1	missense	P174S	no
YIL090W	ICE2	missense	C364R	yes
YIL166C	SOA1	indel	394	no
YIL169C	CSS1	indel	942	no

YIR016W	None	missense	D252H	no
YJL005W	CYR1	missense	H1984L	no
YJL080C	SCP160	missense	R152G	no
YJL158C	CIS3	missense	S191N	no
YJR062C	NTA1	missense	S424G	no
YJR109C	CPA2	missense	A1049P	no
YKL021C	MAK11	missense	C252R	no
YKL040C	NFU1	missense	M213I	no
YKL078W	DHR2	missense	G161A	no
YKL080W	VMA5	missense	L130S	no
YKL182W	FAS1	missense	A632T	no
YKL183W	LOT5	missense	D54V	no
YKL191W	DPH2	missense	N386K	no
YKL215C	OXP1	missense	T310A	no
YKR021W	ALY1	missense	L621M	no
YKR039W	GAP1	missense	A527T	no
YKR054C	DYN1	missense	N2915I	no
YKR095W	MLP1	missense	Q1040P	no
YLL061W	MMP1	missense	H550N	no
YLR020C	YEH2	missense	Q479L	no
YLR024C	UBR2	missense	S1400C	no
YLR024C	UBR2	nonsense	S1483*	no
YLR045C	STU2	missense	P146S	no
YLR067C	PET309	missense	G350D	no
YLR096W	KIN2	indel	730	no
YLR145W	RMP1	missense	C132Y	no
YLR196W	PWP1	missense	E129D	yes
YLR332W	MID2	missense	I359L	no
YLR369W	SSQ1	missense	K303R	no
YLR383W	SMC6	missense	M270V	yes
YLR410W	VIP1	missense	K262T	yes
YLR417W	VPS36	missense	A412G	no
YLR422W	DCK1	missense	S370L	no
YLR454W	FMP27	missense	M1621I	no
YML072C	TCB3	missense	D168E	no
YML097C	VPS9	missense	C260Y	no
YML100W	TSL1	missense	M121I	no
YMR026C	PEX12	missense	T241M	no
YMR092C	AIP1	missense	Y515H	no
YMR129W	POM152	missense	G1069S	no
YMR154C	RIM13	missense	A93G	no
YMR178W	None	missense	S234T	no
YMR207C	HFA1	missense	G110D	yes
YMR246W	FAA4	missense	F356C	yes
YMR317W	None	indel	270	yes
YMR317W	None	indel	270	yes
YNL054W	VAC7	missense	P143S	no
YNL077W	APJ1	missense	P107Q	no
YNL078W	NIS1	indel	4	no

YNL082W	PMS1	missense	T92M	no
YNL178W	RPS3	missense	A80T	no
YNL258C	DSL1	missense	K615N	no
YNL261W	ORC5	missense	D323G	yes
YNL287W	SEC21	missense	S405Y	no
YNR016C	ACC1	missense	A1019V	yes
YNR030W	ALG12	missense	N477K	no
YNR059W	MNT4	nonsense	W285*	no
YOL039W	RPP2A	missense	G74A	no
YOL075C	None	missense	C506F	no
YOL081W	IRA2	missense	A1845S	yes
YOL110W	SHR5	missense	R113P	no
YOR076C	SKI7	missense	D430V	no
YOR101W	RAS1	missense	R109T	no
YOR107W	RGS2	missense	H71Q	no
YOR151C	RPB2	missense	G888C	yes
YOR168W	GLN4	missense	F61Y	yes
YOR195W	SLK19	missense	N291S	yes
YOR204W	DED1	missense	H93Q	no
YOR241W	MET7	missense	E306D	no
YOR275C	RIM20	nonsense	E269*	no
YOR291W	YPK9	missense	E1231V	no
YOR301W	RAX1	missense	Q179H	no
YOR335C	ALA1	missense	K611M	no
YOR354C	MSC6	missense	T562R	no
YPL045W	VPS16	missense	V617I	no
YPL056C	LCL1	missense	V55L	no
YPL086C	ELP3	missense	L512F	no
YPL100W	ATG21	missense	K32N	no
YPL106C	SSE1	missense	L278F	yes
YPL116W	HOS3	missense	Q578P	no
YPL184C	MRN1	missense	E91Q	no
YPL249C	GYP5	missense	M472I	no
YPL264C	None	missense	M273I	no
YPL272C	PBI1	missense	G25R	no
YPR014C	None	missense	V39G	no
YPR029C	APL4	missense	H64N	no
YPR043W	RPL43A	missense	C12S	no
YPR095C	SYT1	missense	Y1058N	no
YPR097W	None	missense	V368L	no
YPR116W	RRG8	nonsense	S86*	no
YPR120C	CLB5	missense	D43N	yes
YPR138C	MEP3	missense	A26P	no
YPR173C	VPS4	missense	E126Q	no
YPR192W	AQY1	missense	F62S	no
