# Supplementary material for: Genetic interactions between specific chromosome copy number alterations dictate complex aneuploidy patterns
Source: Genes Dev. 2018 Dec 1;32(23-24):1485–98. doi: 10.1101/gad.319400.118 (PMC6295164; doi:10.1101/gad.319400.118)
Supplement: Supplemental Material [file supp_gad.319400.118_Supplemental_Table_S4.pdf]

Table S4. List of heterozygous mutations in the *bir1Δ-ad* strains.

Chromosome	Systematic name	Standard name	Mutant type	Residue change
1	YAL048C	GEM1	missense	H356N
1	YAL034C	FUN19	missense	A286S
2	tL(UAA)B1	None	missense	V15F
2	YBL098W	BNA4	missense	G313C
2	YBL079W	NUP170	missense	F669Y
2	YBL066C	SEF1	missense	H42Y
2	YBL063W	KIP1	missense	P1070L
2	YBL061C	SKT5	missense	A378T
2	YBL050W	SEC17	missense	R73M
2	YBL047C	EDE1	missense	R387K
2	YBL022C	PIM1	missense	A851V
2	YBL019W	APN2	missense	F31L
2	YBR045C	GIP1	missense	S233R
2	YBR066C	NRG2	missense	K68Q
2	YBR092C	PHO3	missense	S132P
2	YBR133C	HSL7	missense	S29C
2	YBR136W	MEC1	missense	S1709*
2	YBR162C	TOS1	missense	G449A
2	YBR180W	DTR1	missense	A202V
2	YBR222C	PCS60	missense	S217N
2	YBR236C	ABD1	missense	T313I
2	YBR272C	HSM3	missense	Y73*
2	YBR285W	None	missense	F123L
2	YBR289W	SNF5	missense	D315Y
3	YCR032W	BPH1	missense	H1648Q
3	YCR061W	None	missense	G442A
3	YCR093W	CDC39	missense	V511M
8	YHL030W	ECM29	missense	F1508C
8	YHR070W	TRM5	missense	V30M
8	YHR193C	EGD2	missense	K100N
10	YJL208C	NUC1	missense	L265F
10	YJL165C	HAL5	missense	E20K
10	YJL090C	DPB11	missense	G284D
10	YJL051W	IRC8	missense	N728I
10	YJL039C	NUP192	missense	K567E
10	YJL034W	KAR2	missense	P162T
10	YJR045C	SSC1	missense	K579N
10	YJR052W	RAD7	missense	L444Q
10	YJR094C	IME1	missense	H243N
10	YJR113C	RSM7	missense	E36D
10	YJR117W	STE24	missense	D52N
