# Supplementary material for: Genetic interactions between specific chromosome copy number alterations dictate complex aneuploidy patterns
Source: Genes Dev. 2018 Dec 1;32(23-24):1485–98. doi: 10.1101/gad.319400.118 (PMC6295164; doi:10.1101/gad.319400.118)
Supplement: Supplemental Material [file supp_gad.319400.118_Supplemental_Figure_S1.pdf]

Ravichandran et al. Figure S1

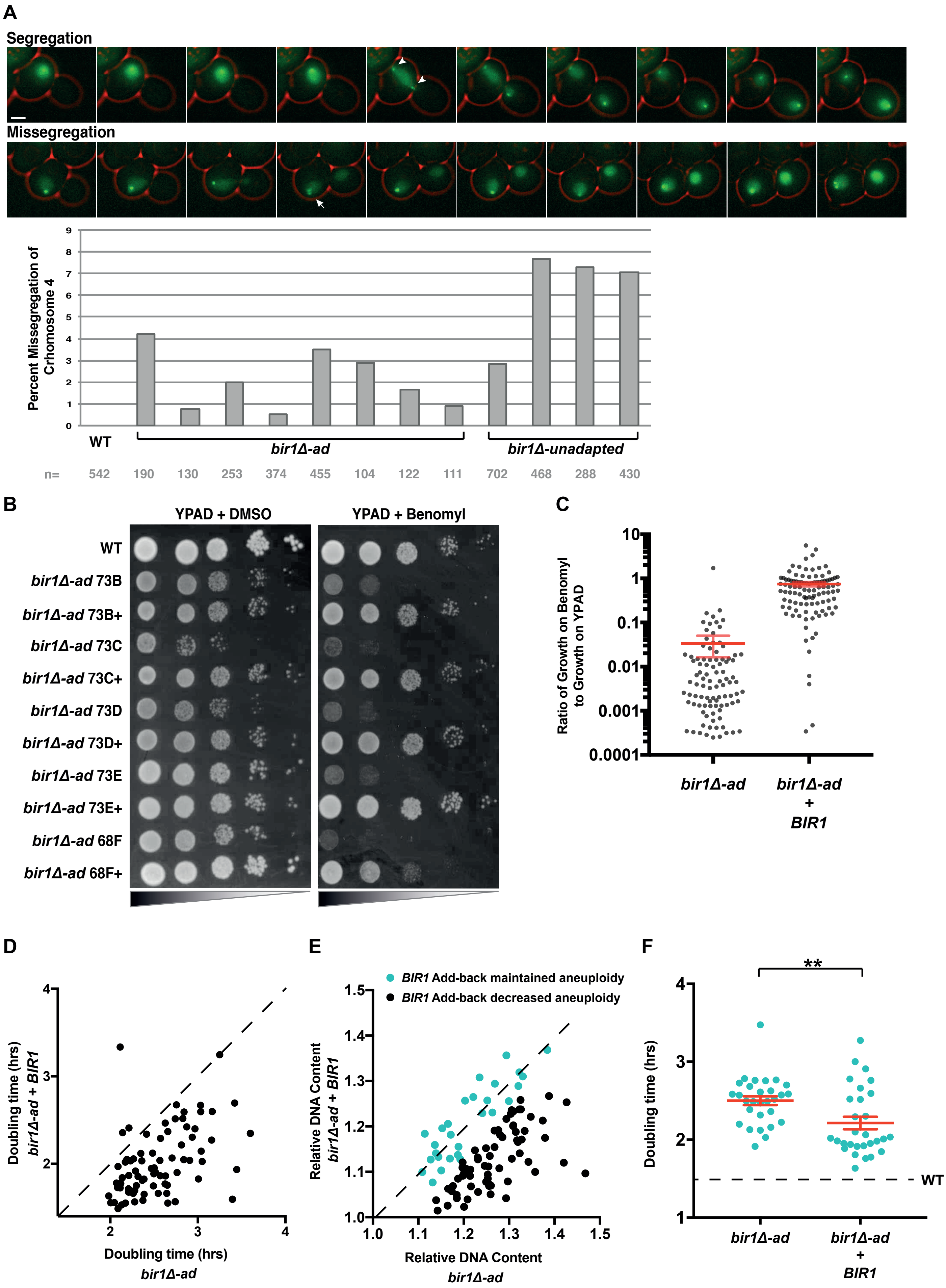

Figure S1: Readdition of *BIR1* to *bir1Δ-ad* strains.

A) Missegregation rates of GFP-labeled chromosome 4 for *bir1Δ-ad* strains. The time lapse images show examples of properly segregated (arrowheads) and missegregated (arrow) chromosome 4. The time interval between images is 6 minutes and the scale bar is 2 μm long. The graph below shows the quantification of the missegregation rates per chromosome of wild-type (WT), eight different *bir1Δ-ad* strains, and four different unadapted *bir1Δ* strains. No missegregation events were observed for the wild-type cells. The total number of segregation events quantified (n) are indicated below the graph.
B) Sensitivity to a moderate (10 μg/ml) amount of the microtubule-depolymerizing drug Benomyl in *bir1Δ-ad* and *bir1Δ-ad* + *BIR1* add-back strains. 10-fold dilutions spotted for wildtype and 5 representative *bir1Δ-ad* and their respective *BIR1* add-back strains on YPAD + DMSO plates with and without Benomyl.
C) Ratio of growth for all 102 *bir1Δ-ad* and *bir1Δ-ad* + *BIR1* with vs. without Benomyl. The mean values and the standard errors are shown in red.
D) Plot of the doubling times for *bir1Δ-ad* strains vs. *bir1Δ-ad* + *BIR1* add-back strains.
E) Plot of the DNA content as measured by flow cytometry for *bir1Δ-ad* strains vs. *bir1Δ-ad* + *BIR1* add-back strains. The blue dots indicate strains that maintained a similar degree of aneuploidy after the add-back of *BIR1*.
F) Doubling times of *bir1Δ-ad* and *bir1Δ-ad* + *BIR1* add-back strains for strains that maintained similar amounts of aneuploidy (blue dots from panel **E)** after the readdition of *BIR1*. Doubling times were measured by optical density. The mean values and the standard errors are shown in red. ** $p < 0.001$.
