# Supplementary material for: Genetic interactions between specific chromosome copy number alterations dictate complex aneuploidy patterns
Source: Genes Dev. 2018 Dec 1;32(23-24):1485–98. doi: 10.1101/gad.319400.118 (PMC6295164; doi:10.1101/gad.319400.118)
Supplement: Supplemental Material [file supp_gad.319400.118_Supplemental_Figure_S5.pdf]

A

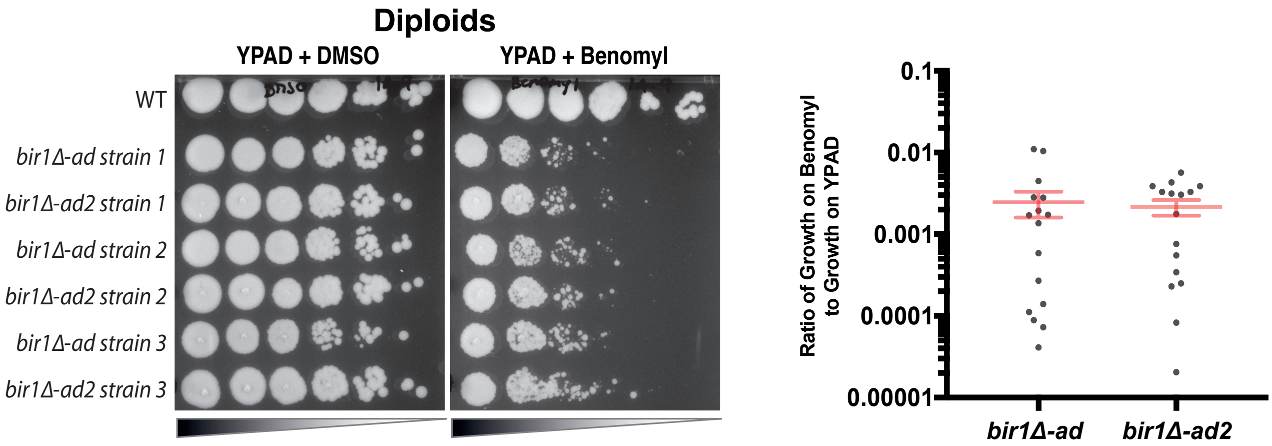

B

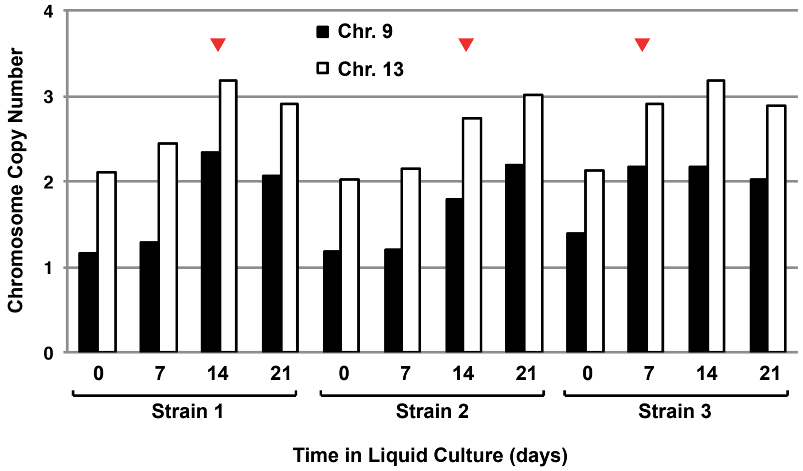

Figure S5: Further of adaptation in liquid media for three diploid *bir1Δ-ad* strains.

A) Sensitivity to a moderate (10 μg/ml) amount of the microtubule-depolymerizing drug Benomyl in *bir1Δ-ad* and *bir1Δ-ad2* strains (Further adapted for 21 days). 10-fold dilutions spotted for wildtype and 3 representative *bir1Δ-ad* and their respective *bir1Δ-ad2* strains on YPAD + DMSO plates with and without Benomyl. Mean values and the standard errors are in red.
B) The copy number of chromosome 9 (black) and 13 (white) for 3 *bir1Δ-ad* strains after 0, 7, 14, and 21 days of further liquid adaptation. The chromosome copy numbers on the 0 and 21 days are from relative read counts in whole-genome sequencing data while those for the 7 and 14 day time points are from qPCR. Red arrowheads signify the point at which both chromosomes 9 and 13 gained copies.
