# Supplementary material for: Genetic interactions between specific chromosome copy number alterations dictate complex aneuploidy patterns
Source: Genes Dev. 2018 Dec 1;32(23-24):1485–98. doi: 10.1101/gad.319400.118 (PMC6295164; doi:10.1101/gad.319400.118)
Supplement: Supplemental Material [file supp_gad.319400.118_Supplemental_Figure_S2.pdf]

Ravichandran et al. Figure S2

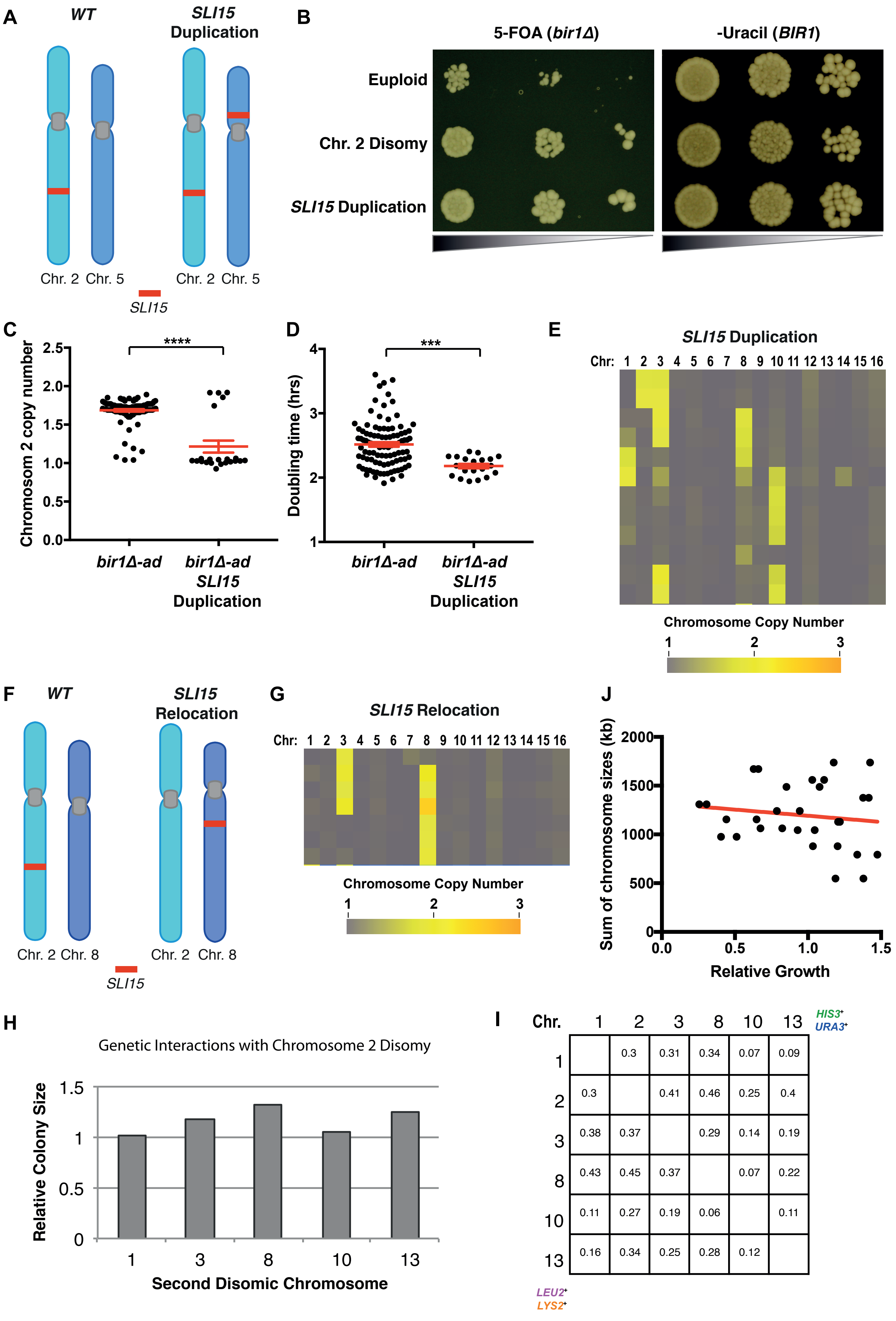


| Chr. | 1 | 2 | 3 | 8 | 10 | 13 |
|---|---|---|---|---|---|---|
| 1 | | 0.3 | 0.31 | 0.34 | 0.07 | 0.09 |
| 2 | 0.3 | | 0.41 | 0.46 | 0.25 | 0.4 |
| 3 | 0.38 | 0.37 | | 0.29 | 0.14 | 0.19 |
| 8 | 0.43 | 0.45 | 0.37 | | 0.07 | 0.22 |
| 10 | 0.11 | 0.27 | 0.19 | 0.06 | | 0.11 |
| 13 | 0.16 | 0.34 | 0.25 | 0.28 | 0.12 | |

**Figure S2: Chromosome 2 disomy contributes to adaptation to *BIR1* deletion by increasing the copy number of the *SLI15* gene.**

**A)** Schematic for the duplication of the *SLI15* gene. One copy of *SLI15* was added to chromosome 5.
**B)** 10-fold dilution series of strains on 5-FOA (selecting against *URA3*) and -uracil (selecting for *URA3*) plates. Selection against *URA3* eliminates the copy of *BIR1* present on a minichromosome, resulting in *bir1Δ* cells.
**C)** Chromosome 2 copy numbers in *bir1Δ-ad* strains and *bir1Δ-ad* strains with *SLI15* duplication prior to adaptation as measured by qPCR. Mean values and the standard errors are shown in red.
**D)** Doubling times for *bir1Δ-ad* strains and *bir1Δ-ad* strains with *SLI15* duplication prior to adaptation as measured by optical density in rich liquid media. Mean values and the standard errors are shown in red.
**E)** Heat-map visualization of the chromosome copy numbers for the *bir1Δ-ad* + *SLI15* duplication strains. Each of the strains is represented as a row.
**F)** Schematic for the relocation of the *SLI15* gene, with the sole copy of *SLI15* now on chromosome 8.
**G)** Heat-map visualization of the chromosome copy numbers for the *bir1Δ-ad* + *SLI15* relocation strains. Each of the strains is represented as a row.
**H)** Relative colony sizes for strains that contain an extra copy of chromosome 2 and one of the other indicated chromosomes. Values were normalized as in Figure **3E**.
**I)** Median colony sizes in square millimeters of double disomic strains on YPAD plates. Normalized values are in Figure **3E**.
**J)** Scatter plot of relative colony sizes obtained from Figures **3E** and **S2H** plotted against the sum of the chromosome sizes of the two induced disomic chromosomes (r = -0.1368, p = 0.48).
