# Supplementary material for: Genetic interactions between specific chromosome copy number alterations dictate complex aneuploidy patterns
Source: Genes Dev. 2018 Dec 1;32(23-24):1485–98. doi: 10.1101/gad.319400.118 (PMC6295164; doi:10.1101/gad.319400.118)
Supplement: Supplemental Material [file supp_gad.319400.118_Supplemental_Figure_S4.pdf]

Ravichandran et al. Figure S4

Colon Adenocarcinoma

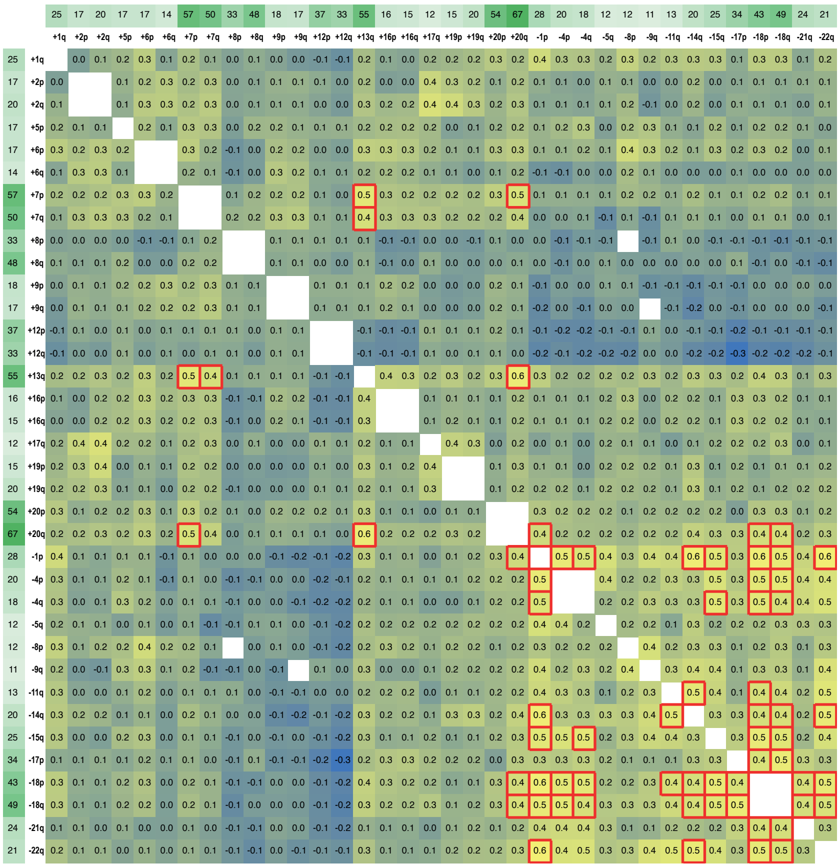

Glioblastoma Multiforme

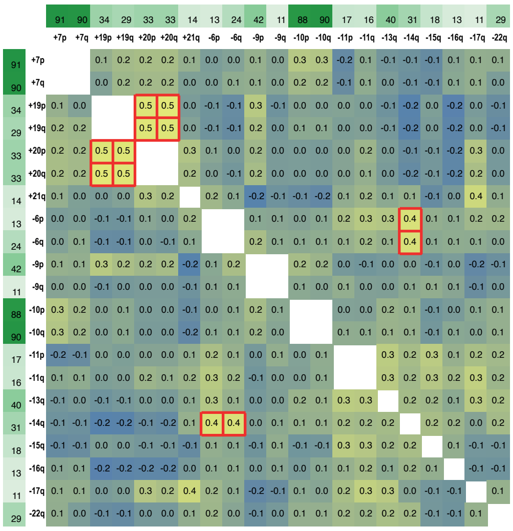

Kidney Renal Clear Cell Carcinoma

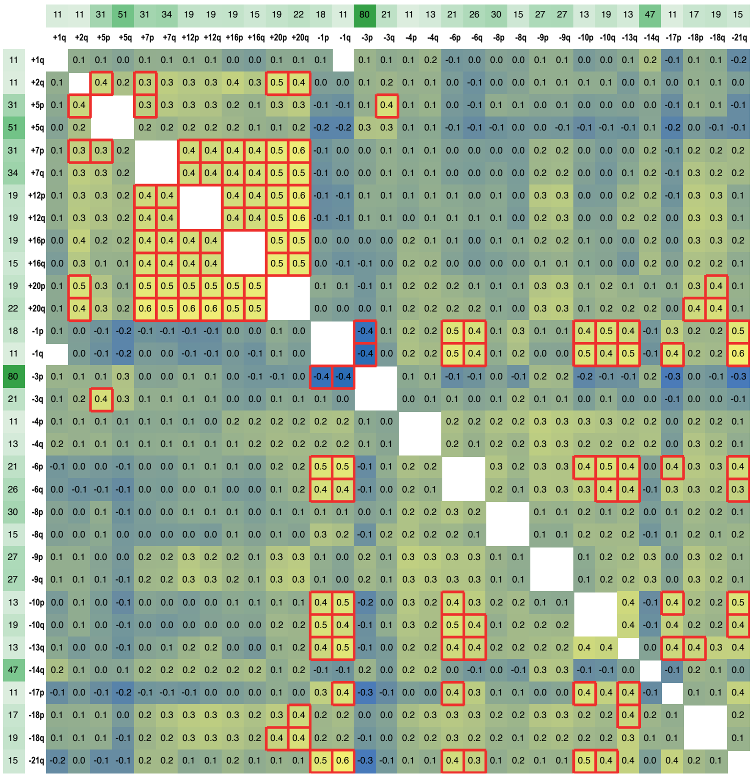

Stomach Adenocarcinoma

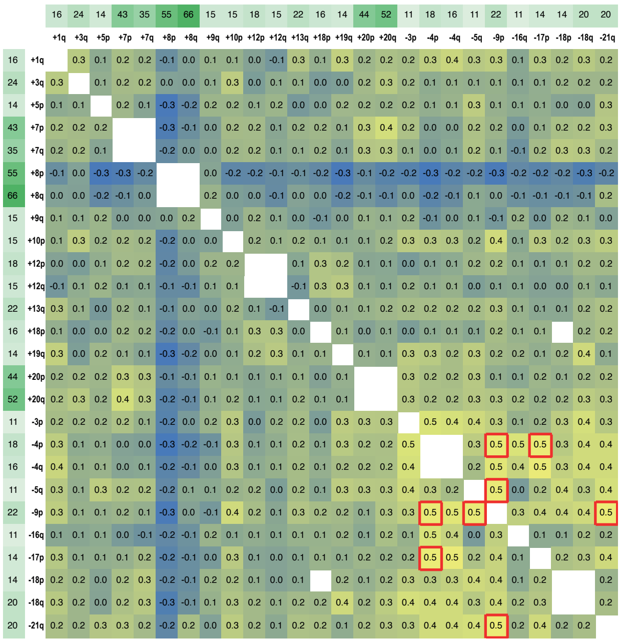

-0.6 Correlation 0.6

P < 5 x 10^{-7}

Figure S4: Chromosome arm copy number correlations in four different cancer types.

Pairwise correlation coefficients for chromosome arm aneuploidy are shown as a heat map for 138 Colon Adenocarcinoma, 160 Glioblastoma Multiforme, 253 Kidney Renal Clear Cell Carcinoma, and 119 Stomach Adenocarcinoma tumors. The frequency of each aberration is shown in green as a percentage. The red highlighted border indicates a P < 5 x 10-7 in the hypergeometric test.
