# Supplementary material for: Genetic interactions between specific chromosome copy number alterations dictate complex aneuploidy patterns
Source: Genes Dev. 2018 Dec 1;32(23-24):1485–98. doi: 10.1101/gad.319400.118 (PMC6295164; doi:10.1101/gad.319400.118)
Supplement: Supplemental Material [file supp_gad.319400.118_Supplemental_Figure_S3.pdf]

Ravichandran et al. Figure S3

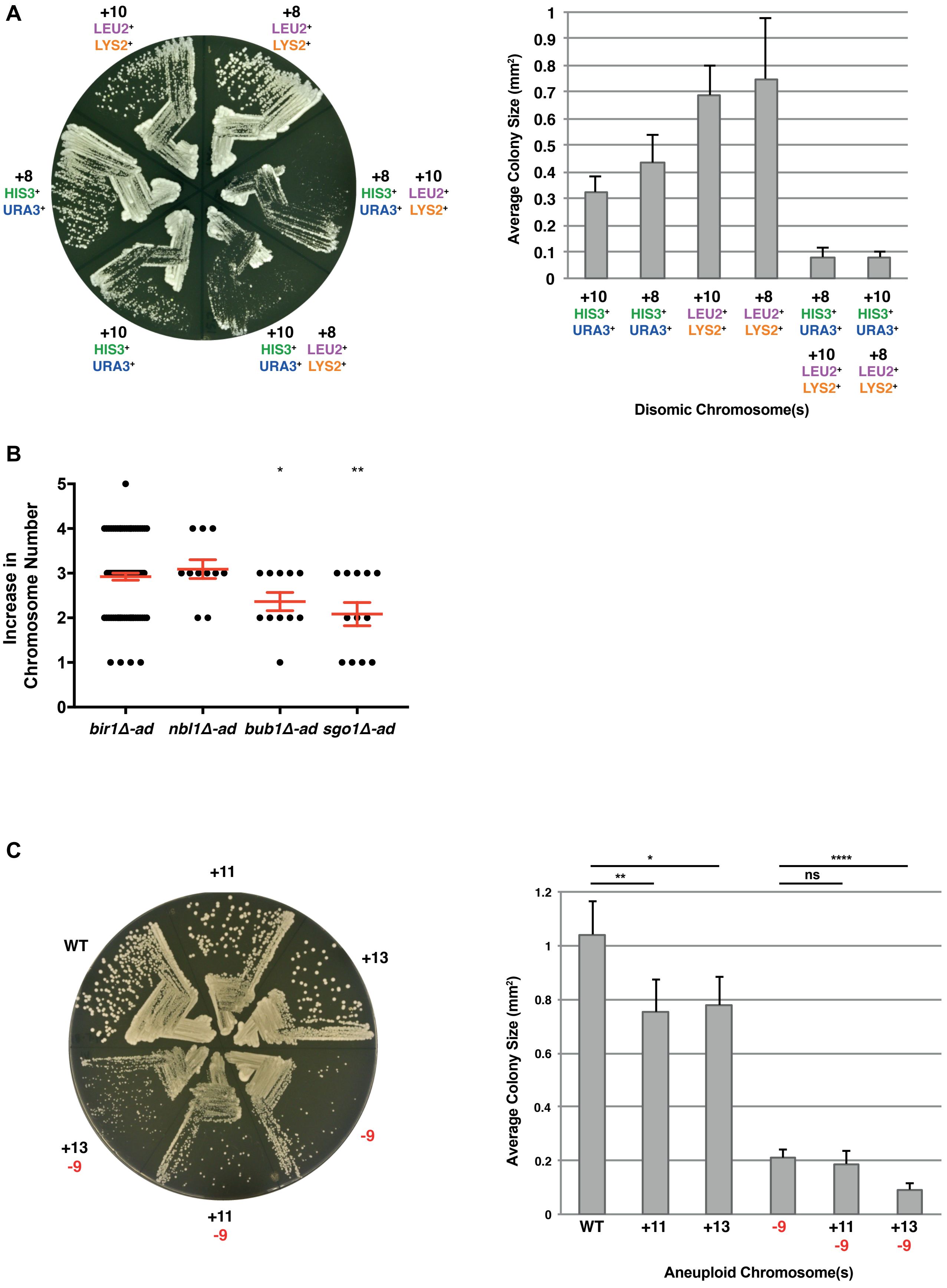

Figure S3: Synthetic negative genetic interaction between chromosome 8 and 10 disomy and growth rates for different diploid *bir1Δ-ad* karyotypes.

A) Strains with disomy of chromosomes 8, 10, or both were struck out for single colonies on a YPAD plate. The graph on the right shows the measured colony sizes (mm^2). Error bars represent the standard deviation for average colony sizes of three independently generated strains (n = 3).
B) Increase in the number of total chromosomes for adapted strains with the indicated mutations. Mean values and the standard errors are in red. $*p \leq 0.05$, $**p < 0.001$.
C) Strains with either aneuploidy of chromosomes 13, 11, 9 or a combination of 11 and 9 or 13 and 9 were struck out for single colonies on a YPAD plate. The graph on the right shows the measured colony sizes. Error bars represent the standard deviation for average colony sizes for 2 replicates of three independently generated strains (n = 6). $*p \leq 0.05$, $**p < 0.001$, $***p < 0.0001$.
