# Supplementary material for: Genetic interactions between specific chromosome copy number alterations dictate complex aneuploidy patterns
Source: Genes Dev. 2018 Dec 1;32(23-24):1485–98. doi: 10.1101/gad.319400.118 (PMC6295164; doi:10.1101/gad.319400.118)
Supplement: Supplemental Material [file supp_gad.319400.118_Supplemental_Table_S1.pdf]

Table S1. Plasmids and Yeast Strains used in this study.

Strain	Genotype	Source	Background	Figure(s)
CCY747	MATα, leu2,3-112, lys2Δ, ura3-1, his3-11:pCUP1-GFP12-lacI12:HIS3, trp1-1:256lacO:TRP1	a	W303	1B
CCY149	MATa/MATα, ura3-1/ura3-1, LEU2/leu2,3-112, LYS2/lys2Δ, ADE2/ade2-1, can1-100, bar1Δ, his3-11:pCUP1-GFP12-lacI12:HIS3/his3-11:pCUP1-GFP12-lacI12:HIS3 trp1-1:256lacO:TRP1/ trp1-1:256lacO:TRP1, BIR1/bir1Δ::hphNT1	a	W303	1
bir1Δ-ad haploids	All 102 strains were derived from tetrad dissection of CCY149	c	W303	1B, 1C, 1D, 1E, 3A, 3B, 3C, 3F, S1
bir1Δ-ad2 haploids	All haploid strains were derived from bir1Δ-ad haploids	c	W303	7B, 7C
BY4741	MATa, his3Δ1, leu2Δ0, ura3Δ0, met15Δ0	b	S288c	-
BY4742	MATα, his3Δ1, leu2Δ0, ura3Δ0	b	S288c	-
CCY1865	MATa, his3Δ1, leu2Δ0, ura3Δ0	c	S288c	-
CCY1905	MATa, leu2,3-112, lys2Δ, ura3-1, his3-11:pCUP1-GFP12-lacI12:HIS3, trp1-1:256lacO:TRP1, bir1Δ::hphNT1, pCC598:URA3	c	W303	-
CCY1976	CCY1905 + cen2::p-$_{GAL1}$-CEN3:LYS2/cen2::p-$_{GAL1}$-CEN3:lys2::pCC644:LEU2	c	W303	2B
CCY1947	CCY1905 + cen10::p-$_{GAL1}$-CEN3:LYS2/cen10::p-$_{GAL1}$-CEN3:lys2::pCC644:LEU2	c	W303	2B
CCY2161	CCY1905 + cen3::p-$_{GAL1}$-CEN3:LYS2/cen3::p-$_{GAL1}$-CEN3:lys2::pCC644:LEU2	c	W303	2B
CCY1943	CCY1905 + cen8::p-$_{GAL1}$-CEN3:LYS2/cen8::p-$_{GAL1}$-CEN3:lys2::pCC644:LEU2	c	W303	2B
CCY2284	CCY1905 + cen9::p-$_{GAL1}$-CEN3:LYS2/cen9::p-$_{GAL1}$-CEN3:lys2::pCC644:LEU2	c	W303	2B
CCY1924	CCY1865 + cen8::p-$_{GAL1}$-CEN3:URA3/cen8::p-$_{GAL1}$-CEN3:ura3::pCC644:HIS3	c	S288c	S3A
CCY1927	CCY1865 + cen10::p-$_{GAL1}$-CEN3:URA3/cen10::p-$_{GAL1}$-CEN3:ura3::pCC644:HIS3	c	S288c	S3A
CCY2122	CCY1865 + cen10::p-$_{GAL1}$-CEN3:LYS2/cen10::p-$_{GAL1}$-CEN3:lys2::pCC644:LEU2	c	S288c	S3A
CCY2125	BY4742 + cen8::p-$_{GAL1}$-CEN3:LYS2/cen8::p-$_{GAL1}$-CEN3:lys2::pCC644:LEU2	c	S288c	S3A
CCY2073	BY4742 + cen3::p-$_{GAL1}$-CEN3:URA3/cen3::p-$_{GAL1}$-CEN3:ura3::pCC644:HIS3, cen8::p-$_{GAL1}$-CEN3:LYS2/cen8::p-$_{GAL1}$-CEN3:lys2::pCC644:LEU2	c	S288c	3E, 3F
CCY2074	BY4742 + cen2::p-$_{GAL1}$-CEN3:URA3/cen2::p-$_{GAL1}$-CEN3:ura3::pCC644:HIS3, cen8::p-$_{GAL1}$-CEN3:LYS2/cen8::p-$_{GAL1}$-CEN3:lys2::pCC644:LEU2	c	S288c	S2H
CCY2075	BY4742 + cen3::p-$_{GAL1}$-CEN3:URA3/cen3::p-$_{GAL1}$-CEN3:ura3::pCC644:HIS3, cen10::p-$_{GAL1}$-CEN3:LYS2/cen10::p-$_{GAL1}$-CEN3:lys2::pCC644:LEU2	c	S288c	3E, 3F

CCY2076	BY4742 + cen10::p-$_{GAL1}$-CEN3:URA3/cen10::p-$_{GAL1}$-CEN3:ura3::pCC644:HIS3, cen8::p-$_{GAL1}$-CEN3:LYS2/cen8::p-$_{GAL1}$-CEN3:lys2::pCC644:LEU2	c	S288c	3E, 3F
CCY2077	BY4742 + cen2::p-$_{GAL1}$-CEN3:URA3/cen2::p-$_{GAL1}$-CEN3:ura3::pCC644:HIS3, cen10::p-$_{GAL1}$-CEN3:LYS2/cen10::p-$_{GAL1}$-CEN3:lys2::pCC644:LEU2	c	S288c	S2h
CCY2078	BY4742 + cen8::p-$_{GAL1}$-CEN3:URA3/cen8::p-$_{GAL1}$-CEN3:ura3::pCC644:HIS3, cen10::p-$_{GAL1}$-CEN3:LYS2/cen10::p-$_{GAL1}$-CEN3:lys2::pCC644:LEU2	c	S288c	3E, 3F
CCY2260	BY4741 + cen1::p-$_{GAL1}$-CEN3:URA3/cen1::p-$_{GAL1}$-CEN3:ura3::pCC644:HIS3, cen8::p-$_{GAL1}$-CEN3:LYS2/cen8::p-$_{GAL1}$-CEN3:lys2::pCC644:LEU2	c	S288c	3E, 3F
CCY2261	BY4741 + cen1::p-$_{GAL1}$-CEN3:URA3/cen1::p-$_{GAL1}$-CEN3:ura3::pCC644:HIS3, cen10::p-$_{GAL1}$-CEN3:LYS2/cen10::p-$_{GAL1}$-CEN3:lys2::pCC644:LEU2	c	S288c	3E, 3F
CCY2264	BY4741 + cen3::p-$_{GAL1}$-CEN3:URA3/cen3::p-$_{GAL1}$-CEN3:ura3::pCC644:HIS3, cen8::p-$_{GAL1}$-CEN3:LYS2/cen8::p-$_{GAL1}$-CEN3:lys2::pCC644:LEU2	c	S288c	3E, 3F
CCY2266	BY4742 + cen13::p-$_{GAL1}$-CEN3:URA3/cen13::p-$_{GAL1}$-CEN3:ura3::pCC644:HIS3, cen10::p-$_{GAL1}$-CEN3:LYS2/cen10::p-$_{GAL1}$-CEN3:lys2::pCC644:LEU2	c	S288c	3E, 3F
CCY2364	BY4742 + cen13::p-$_{GAL1}$-CEN3:URA3/cen13::p-$_{GAL1}$-CEN3:ura3::pCC644:HIS3, cen3::p-$_{GAL1}$-CEN3:LYS2/cen3::p-$_{GAL1}$-CEN3:lys2::pCC644:LEU2	c	S288c	3E, 3F
CCY2366	BY4741 + cen2::p-$_{GAL1}$-CEN3:URA3/cen2::p-$_{GAL1}$-CEN3:ura3::pCC644:HIS3, cen3::p-$_{GAL1}$-CEN3:LYS2/cen3::p-$_{GAL1}$-CEN3:lys2::pCC644:LEU2	c	S288c	S2H
CCY2368	BY4742 + cen8::p-$_{GAL1}$-CEN3:URA3/cen8::p-$_{GAL1}$-CEN3:ura3::pCC644:HIS3, cen3::p-$_{GAL1}$-CEN3:LYS2/cen3::p-$_{GAL1}$-CEN3:lys2::pCC644:LEU2	c	S288c	3E, 3F
CCY2369	BY4741 + cen10::p-$_{GAL1}$-CEN3:URA3/cen10::p-$_{GAL1}$-CEN3:ura3::pCC644:HIS3, cen3::p-$_{GAL1}$-CEN3:LYS2/cen3::p-$_{GAL1}$-CEN3:lys2::pCC644:LEU2	c	S288c	3E, 3F
CCY2370	BY4742 + cen13::p-$_{GAL1}$-CEN3:URA3/cen13::p-$_{GAL1}$-CEN3:ura3::pCC644:HIS3, cen3::p-$_{GAL1}$-CEN3:LYS2/cen3::p-$_{GAL1}$-CEN3:lys2::pCC644:LEU2	c	S288c	3E, 3F
CCY2372	BY4741 + cen2::p-$_{GAL1}$-CEN3:URA3/cen2::p-$_{GAL1}$-CEN3:ura3::pCC644:HIS3, cen1::p-$_{GAL1}$-CEN3:LYS2/cen1::p-$_{GAL1}$-CEN3:lys2::pCC644:LEU2	c	S288c	S2H
CCY2374	BY4741 + cen3::p-$_{GAL1}$-CEN3:URA3/cen3::p-$_{GAL1}$-CEN3:ura3::pCC644:HIS3, cen1::p-$_{GAL1}$-	c	S288c	3E, 3F

	CEN3:LYS2/cen1::p-$_{GAL1}$-CEN3:lys2::pCC644:LEU2			
CCY2376	*BY4741 + cen8::p-$_{GAL1}$-CEN3:URA3/cen8::p-$_{GAL1}$-CEN3:ura3::pCC644:HIS3, cen1::p-$_{GAL1}$-CEN3:LYS2/cen1::p-$_{GAL1}$-CEN3:lys2::pCC644:LEU2*	c	S288c	3E, 3F
CCY2378	*BY4742 + cen10::p-$_{GAL1}$-CEN3:URA3/cen10::p-$_{GAL1}$-CEN3:ura3::pCC644:HIS3, cen1::p-$_{GAL1}$-CEN3:LYS2/cen1::p-$_{GAL1}$-CEN3:lys2::pCC644:LEU2*	c	S288c	3E, 3F
CCY2379	*BY4742 + cen13::p-$_{GAL1}$-CEN3:URA3/cen13::p-$_{GAL1}$-CEN3:ura3::pCC644:HIS3, cen1::p-$_{GAL1}$-CEN3:LYS2/cen1::p-$_{GAL1}$-CEN3:lys2::pCC644:LEU2*	c	S288c	3E, 3F
CCY1480	*MATa/MATα, ura3-1/ura3-1, LEU2/leu2,3-112, LYS2/lys2Δ, ADE2/ade2-1, can1-100, bar1Δ, his3-11:pCUP1-GFP12-lacI12:HIS3/his3-11:pCUP1-GFP12-lacI12:HIS3 trp1-1:256lacO:TRP1/ trp1-1:256lacO:TRP1, NBL1/nbl1Δ::hphNT1*	c	W303	-
nbl1Δ-ad haploids	*All nbl1Δ-ad strains were derived from tetrad dissection of CCY1480*	c	W303	4B, 4C, 4D, 4E
CCY1482	*MATa/MATα, ura3-1/ura3-1, LEU2/leu2,3-112, LYS2/lys2Δ, ADE2/ade2-1, can1-100, bar1Δ, his3-11:pCUP1-GFP12-lacI12:HIS3/his3-11:pCUP1-GFP12-lacI12:HIS3 trp1-1:256lacO:TRP1/ trp1-1:256lacO:TRP1, SGO1/sgo1Δ::hphNT1*	c	W303	-
sgo1Δ-ad haploids	*All sgo1Δ-ad strains were derived from tetrad dissection of CCY1482*	c	W303	4B, 4C, 4D, 4E
CCY1484	*MATa/MATα, ura3-1/ura3-1, LEU2/leu2,3-112, LYS2/lys2Δ, ADE2/ade2-1, can1-100, bar1Δ, his3-11:pCUP1-GFP12-lacI12:HIS3/his3-11:pCUP1-GFP12-lacI12:HIS3 trp1-1:256lacO:TRP1/ trp1-1:256lacO:TRP1, BUB1/bub1Δ::hphNT1*	c	W303	-
bub1Δ-ad haploids	*All bub1Δ-ad strains were derived from tetrad dissection of CCY1484*	c	W303	4B, 4C, 4D, 4E
CCY1739	*tetradploid, MATa/MATaΔ::natNT2/Matα/MatαΔ::kanMX4, ura3-1, lys2Δ, LEU2/leu2,3-112, his3-11:pCUP1-GFP12-lacI12:HIS3 trp1-1:256lacO:TRP1 ADE2/ade2-1, can1-100, bar1Δ, BIR1/BIR1/bir1Δ::hphNT1/bir1Δ::hphNT1*	c	W303	-
CCY1743	*MATa/MATα, ura3-1/ura3-1, lys2Δ/lys2Δ, LEU2/leu2,3-112, his3-11:pCUP1-GFP12-lacI12:HIS3, trp1-1:256lacO:TRP1, ADE2/ade2-1, can1-100, bar1Δ, bir1Δ::hphNT1/bir1Δ::natNT2, pCC599::URA3*	c	W303	-
CCY1744	*MATa/MATα, ura3-1/ura3-1, lys2Δ/lys2Δ, LEU2/leu2,3-112, his3-11:pCUP1-GFP12-lacI12:HIS3, trp1-1:256lacO:TRP1, ADE2/ade2-1, can1-100, bar1Δ, bir1Δ::hphNT1/bir1Δ::kanMX4, pCC599::URA3*	c	W303	-
bir1Δ-ad diploids	*All 25 strains were derived from tetrad dissection of strains with the genotype of CCY1739, or from counter-selection of URA3 plasmid of CCY1743,*	c	W303	5, S3B

	CCY1744			
bir1Δ-ad2 diploids	*All bir1Δ-ad2 diploid strains were derived from bir1Δ-ad diploids*	c	W303	7D, 7E
CCY1295	*CCY149 + SLI15/sli15 Δ:natNT2*	c	W303	-
CCY1320	*CCY1295 + ura3-1:pCC411:URA3*	c	W303	-
bir1Δ-ad SLI15 Duplication	*All bir1Δ-ad SLI15 Duplication strains were derived from spores of CCY1320*	c	W303	S2C, S2D, S2E
CCY2761	*CCY1295 + cup1-1:pCC607:URA3*	c	W303	-
bir1Δ-ad SLI15 Relocation	*All bir1Δ-ad SLI15 Relocation strains were derived from spores of CCY2761*	c	W303	S2G
CCY2115	*CCY1905 + trp1-1:pCC272:TRP1*	c	W303	S2B

**Plasmid**	**Description**	**Source**		
pRS306	*pBluescript URA3*	d		
pCC272	*SLI15 + 1kb upstream in pRS304*	c		
pCC283	*BIR1 + 1kb upstream in pRS306*	a		
pKA52	*HIS3 integration plasmid with part of URA3 inserted at the MCS. Can insert HIS3 into a URA3 locus. Used for making disomic strains.*	e		
pGALCEN-JC3-13	*For replacing centromeres with CEN3 under the GAL-10 promoter (URA3)*	e		
pCC658	*For replacing centromeres with CEN3 under the GAL-10 promoter (LYS2)*	c		
pCC644	*LEU2 integration vector with bases 3043-3538 of LYS2 for Disruption of the LYS2 gene. Used for making disomic strains.*	c		
pCC598	*BIR1 + 1kb upstream in pRS306*	c		
pCC411	*SLI15 + 1kb upstream in pRS306*	c		
pCC607	*pCC411 + sequence from Cup1-1 gene for integrating Sli15 plus promoter into that location of chromosome 8*	c		

**Key**	**Source**
a	Campbell and Desai, Nature 497: 118–121 (2013)
b	Brachmann CB et al., Yeast. (1998)
c	This study
d	Sinorski and Hieter Genetics 122: 19-27 (1989)
e	Anders et. al., BMC Genetics (2009)
